# Fragmentation pattern of amides by EI and HRESI: study of protonation sites using DFT-3LYP data†

**DOI:** 10.1039/c7ra00408g

**Published:** 2018-06-12

**Authors:** H. H. Fokoue, J. V. Marques, M. V. Correia, L. F. Yamaguchi, X. Qu, J. Aires-de-Sousa, M. T. Scotti, N. P. Lopes, M. J. Kato

**Affiliations:** Laboratório de Avaliação e Síntese de Substâncias Bioativas (LASSBio®), Instituto de Ciências Biomédicas, Centro das Ciências da Saúde, Universidade Federal do Rio de Janeiro CP 68024 21944-971 Rio de Janeiro Brazil; Ginkgo Bioworks Boston MA 02210 USA; Instituto de Química, Universidade de Brasília CP 04478 70704-970 Brasilia-DF Brazil; Instituto de Química, Universidade de São Paulo Av. Prof. Lineu Prestes, 748, 05508-000 São Paulo-SP Brazil massuojorge@gmail.com; LAQV and REQUIMTE, Departamento de Química, Faculdade de Ciências e Tecnologia, Universidade Nova de Lisboa Caparica 2829-516 Portugal; Pós-Graduação em Produtos Naturais e Sintéticos Bioativos, Universidade Federal da Paraíba João Pessoa 58051-900 Brazil; Departamento de Física e Química, Faculdade de Ciências Farmacêuticas de Ribeirão Preto, Universidade de São Paulo Ribeirão Preto SP 14040-903 Brazil

## Abstract

Amides are important natural products which occur in a few plant families. Piplartine and piperine, major amides in *Piper tuberculatum* and *P. nigrum*, respectively, have shown a typical N–CO cleavage when analyzed by EI-MS or HRESI-MS. In this study several synthetic analogs of piplartine and piperine were subjected to both types of mass spectrometric analysis in order to identify structural features influencing fragmentation. Most of the amides showed an intense signal of the protonated molecule [M + H]^+^ when subjected to both HRESI-MS and EI-MS conditions, with a common outcome being the cleavage of the amide bond (N–CO). This results in the loss of the neutral amine or lactam and the formation of aryl acylium cations. The mechanism of N–CO bond cleavage persists in α,β-unsaturated amides because of the stability caused by extended conjugation. Computational methods determined that the protonation of the piperamides and their derivatives takes place preferentially at the amide nitrogen supporting the dominant the N–CO bond cleavage.

## Abstract 2

The N–CO cleavage of α,β-unsaturated piperamides under EI and ESI is supported by computational studies.

## Graphical abstract

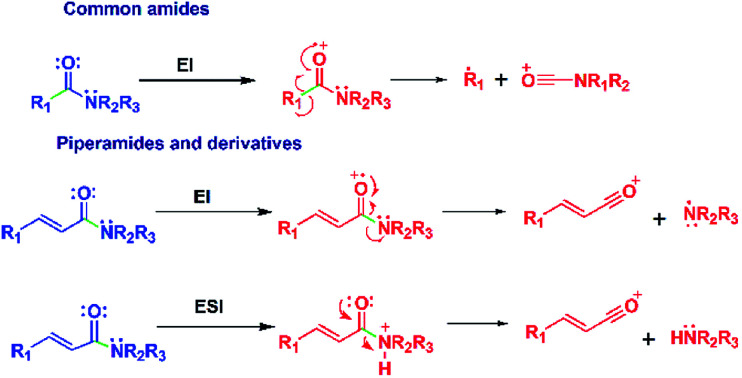

## 1. Introduction

Amides are an important class of natural products found in a few plant families such as Asteraceae, Piperaceae, Rutaceae and Solanaceae, among others.^1–4^ The two most well-known amides are piperine and capsaicin, the pungent principles of black pepper (*Piper nigrum*, Piperaceae) and chili pepper (*Capsicum* varieties, Solanaceae), respectively.^5^ Amides play important roles in plant ecology, being involved in many aspects of plant–insect interactions.^6^ Additionally, they exert many important biological activities. For instance, piplartine, piperine, piperlonguminine, fagaramide, corcovadine and other piperamides have been shown to have cytotoxic, antimicrobial and anti-inflammatory properties.^7–9^ Several amides from different sources have been isolated through bioactivity-guided fractionation of crude extracts. Furthermore, to fully investigate their bioactivity many analogs of naturally occurring amides have been synthesized.^10–12^

Our research group has focused on the phytochemistry of the genus *Piper* because of its wide occurrence in the tropics, ease of propagation, ecological importance and most importantly due to the previously described bioactivity of both crude extracts and isolated compounds from this genus. Thus, the development of fast and reliable tools for rapid dereplication and identification of major compounds in crude extracts became an important issue.

**Fig. 1:**
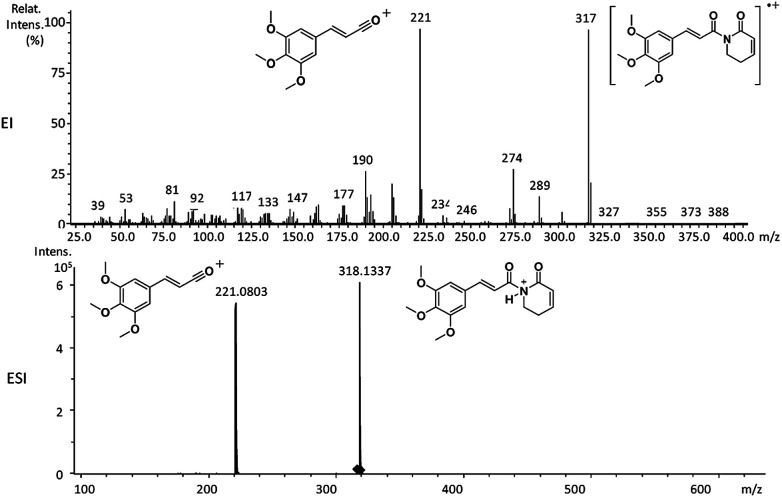
EI-MS and HRESI-MS of piplartine (1a).

**Fig. 2:**
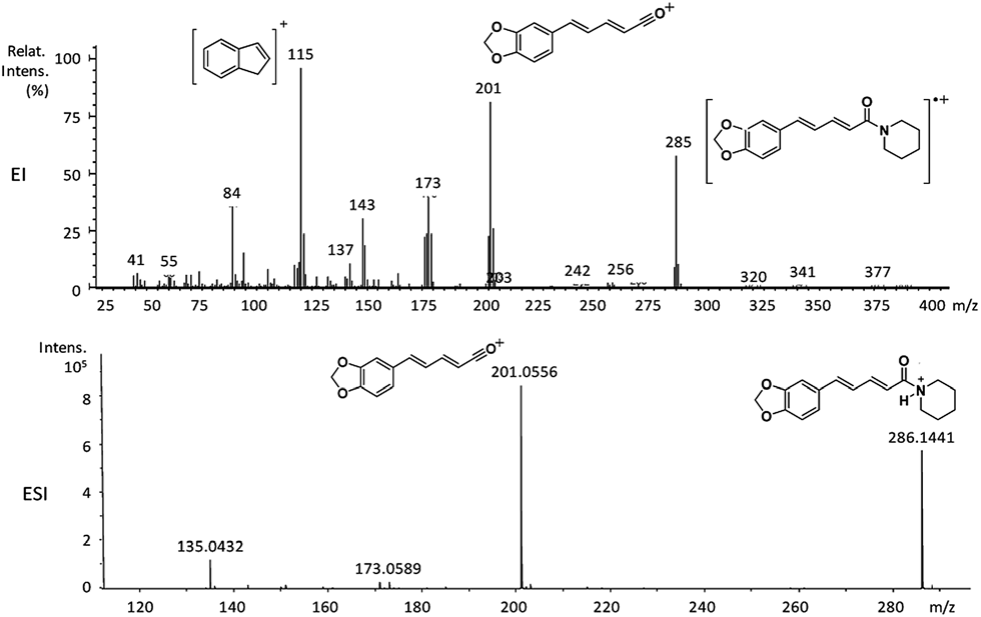
EI-MS and HRESI-MS of piperine (6a).

The application of mass spectrometry based on electron impact has already been applied to determine a series of amides in *P. amalago*.^13^ Two well-known amides, piperine and piplartine, have shown the cleavage of the N–CO bond, a specific fragmentation pattern observed by EI-MS and HRESI-MS (Fig. 1 and 2).^14–16^ This peculiar cleavage, observed in the α,β-unsaturated piperamides is unusual in aliphatic amides, in which McLafferty rearrangement is preferable wherever γ-hydrogen to the carbonyl is available. Additionally, the α-cleavage of secondary and tertiary aliphatic amides and the formation of acylium cations can also be used to support the characterization of amides. The conjugation between the amide carbonyl and the β double bond reinforced by the presence of an aryl group at the γ or 5 positions from the carbonyl could account for preferable N–CO fragmentation.^15,16^ Herein, several analogs synthesized were subjected to mass spectrometry studies to determine whether the N–CO α-cleavage could be generalized and used as a criteria for the rapid identification of piperamides. Computational studies (proton affinity and bond energies) were carried out to verify the N–CO α-cleavage. Both EI and HRESI-MS experiments were then carried out to compare the corresponding fragmentation patterns and, together a calculation of energetic profiles at protonation sites were examined to support the characterization of amides.

## 2. Experimental

### 2.1 Isolation of piplartine and piperine

Roots of *Piper tuberculatum* were harvested on the Campus of the University of São Paulo (USP), São Paulo, Brazil. The botanical classification was performed by Dr Elsie Franklin Guimarães (Instituto de Pesquisas Jardim Botânico do Rio de Janeiro). A voucher specimen (Kato-0169) was deposited at the Herbarium of the same institute. For the isolation of piplartine, 100 g of ground dry roots of *P. tuberculatum* were extracted with a mixture of chloroform/methanol 2 : 1 (1 L) for 72 hours. The solvent mixture was dried under reduced pressure using a roto-evaporator to yield a white solid which, after recrystallization in MeOH,^17^ yielded pure piplartine (1a). The identity of piplartine was confirmed by comparison of NMR and MS data with those reported.^18,19^

Seeds of *P. nigrum* (black-pepper) were purchased in a local market in São Paulo. Piperine (6a) was purified from 250 g of dried *P. nigrum* fruits. The fruits were ground to a fine powder and extracted twice with 2 L of chloroform/methanol (2 : 1) v/v. The extract was filtered and concentrated under vacuum using a roto-evaporator. The crude extract was subjected to a silica column chromatography eluted with hexanes/ethyl acetate mixtures of increasing polarity. Fractions containing piperine (6a) were pooled and recrystallized in methanol yielding a yellowish crystal. Piperine was identified by comparison of NMR and MS analysis with reported data.^19,20^

### 2.2 Synthesis of piplartine and piperine derivatives

Compounds 9 and 10 were obtained by catalytic hydrogenation (4 atm of hydrogen, Pd–C) of piplartine (1a) and piperine (6a), respectively, for 12 hours.^21^

To a solution of 1 equivalent of carboxylic acids in dry THF (10 mL) [*i.e.* 4-bromocinnamic acid; 5-(4-bromophenyl)-(2*E*,4*E*)-2,4-pentadienoic acid; (*E*)-cinnamic acid; 5-(4-hydroxyphenyl)-(2*E*,4*E*)-2,4-pentadienoic acid; 3,4-(methylenedioxy)-cinnamic acid or piperinic acid; (*E*)-3,4,5-trimethoxycinnamic acid; (*E*)-3,4-dimethoxycinnamic acid], kept under nitrogen atmosphere and over ice bath, oxalyl chloride (5 equiv.) was added dropwise and stirred for 6 hours. After reaching room temperature, the excess of oxalyl chloride was removed under reduced pressure to leave the corresponding acyl chlorides. Compounds 1c, 1d–1f, 2a–2d, 3a–3c, 4a, 4b, 5a–5c, 6b, 7a–7c, 8a and 8b were synthesized by addition of triethylamine (3 equiv.) and the appropriate amines (*i.e*. *n*-dibutylamine, morpholine, *n*-pentylamine or piperidine) to a methylene chloride solution of the various acyl chlorides (1.0 equivalent). The reaction mixtures were stirred overnight at room temperature, quenched with saturated aqueous ammonium chloride solution and then extracted with methylene chloride 3 times. Combined organic phases were washed with brine and dried over anhydrous magnesium sulphate. After filtration and concentration, the residues were purified by flash chromatography over silica-gel using the hexanes-ethyl acetate (typically 10–30%) as eluent yielding the desired amides.^15^

Compounds 1b, 2e and 5d were synthesized by the slow addition of *n*-butyl lithium (1.3 equivalent), at −78 °C under nitrogen atmosphere, to a solution of 2-pyrrolidinone (1.2 equiv.) in dry THF. After 15 min, solutions of the corresponding acyl chlorides (1.0 equiv.) were added. The reaction mixtures were stirred for 1 hour at room temperature, quenched with saturated aqueous ammonium chloride solution and then extracted with ethyl acetate (3 times). Combined organic phases were washed with brine and dried over anhydrous magnesium sulphate. After filtration and concentration, the residues were purified by flash chromatography over silica-gel using the hexanes-ethyl acetate as eluent yielding the desired imides.

### 2.3 Mass spectrometry instrumentation (EI-MS and HRESI-MS)

GCMS analysis was performed using a GCMS-QP2010 Ultra gas chromatograph (Shimadzu) with an AOC-20is series injector/autosampler (Shimadzu, Kyoto, Japan) operating in the EI mode at 70 eV with a Rxi-5ms fused silica capillary column (30 m × 0.25 mm ID × 0.25 μm df). The front inlet temperature was 280 °C. The helium gas flow rate through the column was 0.51 mL min^−1^. The column temperature was held isothermally at 60 °C for 1 min and then ramped up from 60 to 320 °C by 35 °C min^−1^ and held isothermally for 6 min. The transfer line and ion-source temperatures were 240 and 260 °C, respectively. Experiments were recorded on scan mode over the mass range 35–500 *m*/*z*.

For HRESI-MS, the samples were analyzed by liquid chromatography and mass spectrometry Bruker MicrOTOF-QII Bruker. The High Performance Liquid Chromatograph (Shimadzu, Kyoto, Japan) was composed by two analytical pumps (model LC-20AD), an automatic injector (SIL-20AHT), a UV/Vis detector (SPD-20A), and a column oven (CTO-20A) controlled by a CBM-20A module. The mobile phase contained acetonitrile (+0.1% of formic acid) v/v and water (+0.1% of formic acid) v/v and the gradient was 40% acetonitrile at 0 min (for up to 2 min), and then it was linearly raised to 100% acetonitrile (from 2 to 30 min) and kept at a plateau for 5 min. The column was a reverse phase Luna 5μ PFP(2) 100 Å, 150 × 2 mm (Phenomenex). The monitored wavelength was 254 nm, and the column oven was set at 40 °C and the mobile phase flow was 200 μL min^−1^ directly infused into the mass spectrophotometer.

The mass spectrophotometer worked in positive mode with N_2_ as nebulizer and drying gas at 4 Bar and 8 L min^−1^, respectively. The drying temperature was set to 200 °C; the collision energy and the quadruple energy were set to 12 and 6 eV, respectively. RF1 and RF2 funnels were programmed to 400 V_pp_ and the monitored mass range was 100–1000 kDa.

### 2.4 NMR analysis

^1^H and ^13^C NMR spectra were acquired using a Varian Gemini 200 spectrometer operating at 200 and 50 MHz, and a Bruker (DRX 300) spectrometer operating at 300 MHz and 75 MHz, both available at the Analytical Center of the Institute of Chemistry of the University of São Paulo.

### 2.5 Computational method

Density functional theory (DFT) calculations were performed using Spartan 10 for Windows (Wavefunction Inc, Irvine, CA, USA).^22,23^ Each studied amide had its neutral, protonated [M + H]^+^ and acyl cation forms examined at the B3LYP/6-311G* level and the lowest energy conformers were selected for the calculations. The global minimum on the potential energy surface was used for the determination of each geometry.

Proton affinity (PA) was defined as the negative of the enthalpy variation (Δ_r_*H*°) for the reaction M + H^+^ → MH^+^:PA = −Δ_r_*H*_298_, Δ_r_*H*_298_ = *E*_el_(MH^+^) − *E*_el_(M).

For H^+^, no calculations are required, and the only other non-zero energy term is the difference in translational energy, which was equal to 3/2 *RT* ≈ 3.7 kJ mol^−1^.^24–26^

Additionally, bond energies were calculated using the Spartan 16 (Wavefunction Inc, Irvine, CA, USA) for windows. The energies of the compounds were calculated with DFT calculations at the level B3LYP/6-311+G** by using the single point method.^27^ Previously a conformational search with the molecular mechanics method MMFF (Merck Molecular Force Field) was performed and the geometry of the lowest energy conformer of each compound was optimized by using the PM6 or AM1 semi-empirical method.^28,29^ Bond energies between amide nitrogen and carbonyl were compared for each compound with protons bound at the amide nitrogen and at the carbonyl oxygen.

## 3. Results and discussion

The 29 amides analyzed in our study were divided in to 10 types primarily, according to the aromatic ring substitution pattern, side chain size and number of double bonds varying from one double bond (1–5), two double bonds (6–8) or saturated chain (9–10) (Table 1 and Fig. 6). Piplartine (1a) and piperine (6a), the model compounds for conjugated amides, were isolated from *Piper* species while their derivatives were synthesized as described. Non-optimized yields of this synthesis ranged from 50 to 90% and all compounds were characterized by NMR and MS analysis (See Experimental).

**Table 1:** Structures of natural amides and derivatives^a^

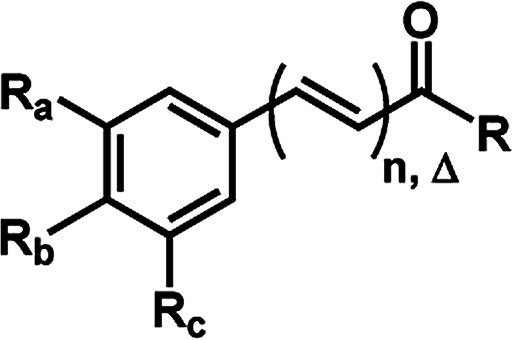					
Amides	*n*	R_a_	R_b_	R_c_	R
1a	1	OMe	OMe	OMe	R_A_
1b	1	OMe	OMe	OMe	R_F_
1c	1	OMe	OMe	OMe	R_E_
1d	1	OMe	OMe	OMe	R_G_
1e	1	OMe	OMe	OMe	R_D_
1f	1	OMe	OMe	OMe	R_C_
2a	1	OMe	OMe	H	R_E_
2b	1	OMe	OMe	H	R_G_
2c	1	OMe	OMe	H	R_D_
2d	1	OMe	OMe	H	R_C_
2e	1	OMe	OMe	H	R_F_
3a	1	H	Br	H	R_D_
3b	1	H	Br	H	R_G_
3c	1	H	Br	H	R_E_
4a	1	OCH_2_O		H	R_D_
4b	1	OCH_2_O		H	R_E_
5a	1	H	H	H	R_E_
5b	1	H	H	H	R_G_
5c	1	H	H	H	R_D_
5d	1	H	H	H	R_F_
6a	2	OCH_2_O		H	R_C_
6b	2	OCH_2_O		H	R_G_
7a	2	H	OMe	H	R_G_
7b	2	H	OMe	H	R_E_
7c	2	H	OMe	H	R_C_
8a	2	H	Br	H	R_G_
8b	2	H	Br	H	R_C_
9	1 (Δ = 0)	OMe	OMe	OMe	R_B_
10	2 (Δ = 0)	OCH_2_O		H	R_C_

a For R see Fig. 6.

Mass spectrometric analysis of amides by EI-MS quite often provides important information for their characterization. In general, aliphatic primary amides produce an intense fragmentary ion peak at *m*/*z* 44 (CONH_2_) resulting from the cleavage of the R–CONH_2_ bond.^30,31^ Aliphatic secondary and tertiary amides having hydrogens at the γ-carbon of the acyl moiety or *N*-methyl groups show intense fragmentary ions resulting from McLafferty rearrangement.^30^ For aromatic amides a resonance-stabilized benzoyl cation is formed, and this may undergo further cleavage of CO loss leading to a phenyl cation.^31^

Piperamides investigated in the present work have aryl groups at the position 3 (1–5) or 5 (6–10) and, are in most of the cases α,β-unsaturated carbonyl amides. These peculiar characteristics prevents the McLafferty rearrangement and lead frequently to the N–CO α-cleavage due to conjugation of the α,β-unsaturated carbonyl group of the amide (Fig. 5). For all amides analyzed under 70 eV, the relatively intense molecular ions [M]˙^+^ were observed (Table 2) and aryl acylium daughter ions were observed for all amides.

**Table 2:** Molecular ions [M]^+^˙ (relative abundance%) and significant fragmentary ions observed in EI-MS spectra of amides^a^

Amide	[M]^+^˙	RCO^+^	Fragmentary ions
1a	317 (90)	221 (100)	274 (32), 193 (20), 190 (32)
1b	305 (100)	221 (55)	205 (40), 190 (22)
1c	349 (37)	221 (100)	222 (63), 190 (15)
1d	307 (85)	221 (95)	236 (42), 222 (100), 191 (26), 190 (27), 181 (55), 179 (27)
1e	307 (50)	221 (100)	222 (60), 190 (26)
1f	305 (60)	221 (65)	222 (100), 194 (25), 191 (35), 190 (25), 84 (69)
2a	319 (20)	191 (100)	276 (12), 262 (10), 163 (11), 151 (15)
2b	277 (43)	191 (100)	206 (35), 192 (40), 151 (45)
2c	277 (31)	191 (100)	192 (23), 163 (14)
2d	275 (55)	191 (100)	192 (35), 163 (20), 161 (20), 84 (42)
2e	275 (51)	191 (100)	163 (12)
3a	295 (21)	209 (72)	211 (70), 183 (13), 181 (11), 126 (27), 102 (100), 86 (52), 56 (34)
3b	295 (10)	209 (74)	211 (75), 102 (100)
3c	337 (5)	209 (100)	211 (91), 102 (100), 44 (83)
4a	261 (57)	175 (100)	176 (32), 145 (81), 117 (37), 89 (47)
4b	303 (18)	175 (100)	145 (42), 89 (26)
5a	259 (7)	131 (100)	216 (14), 103 (33)
5b	217 (7)	131 (100)	188 (10), 146 (20), 103 (45), 77 (25)
5c	217 (18)	131 (100)	103 (60), 86 (26), 77 (28)
5d	215 (26)	131 (100)	187 (14), 103 (57), 77 (32)
6a	285 (63)	201 (87)	202 (25), 173 (42), 143 (35), 115 (100), 84 (35)
6b	287 (55)	201 (57)	173 (99), 115 (100)
7a	273 (87)	187 (100)	188 (35), 155 (20), 159 (62), 144 (60), 121 (42), 115 (60)
7b	315 (26)	187 (100)	144 (27), 128 (27), 115 (27), 44 (35)
7c	271 (67)	187 (100)	159 (30), 144 (40), 115 (42), 84 (35)
8a	321 (18)	235 (37)	237 (35), 156 (25), 128 (100), 96 (45)
8b	319 (25)	235 (25)	237 (25), 156 (27), 138 (27), 129 (28), 128 (100), 84 (74)
9	321 (35)	223 (15)	222 (100), 194 (40), 181 (25), 179 (55), 44 (75)
10	289 (35)	205 (5)	204 (25), 140 (31), 135 (25), 127 (100), 112 (52), 84 (35)

a RCO^+^ = [[M] − NR_d_R_e_]^+^.

The relative intensities of the molecular ions for most of the amides were higher than 10% and quite often ranging from 50–100%. The only exceptions were observed to be 3c (5%) and 5a (7%) and 5b (7%), which were associated to the *N*,*N*-dibutyl and *N*-pentyl amides, respectively. The initial fragmentation yielded acylium ions which were prominent and very informative on the structures of carboxylic acid moieties in all cases. For instance, the acylium ion observed at *m*/*z* 221 for piplartine (1a), associated to the 3,4,5-trimethoxycinnamoyl ion, was also observed for 1b–1f (Table 2 and Fig. 5). Any changes in the substitution pattern of all synthetic amides were accordingly observed in the corresponding acylium ion. Thus, *m*/*z* at 191 (3,4-dimethoxycinnamoyl) was observed for 2a–2e; *m*/*z* at 209 (*p*-bromocinnamoyl) for 3a–3c (Table 2 and Fig. 5). In the cases of amides of types 3 and 8, the typical isotope M + 2 for brominated compounds can be tracked and the acylium ion produces further the peak base at *m*/*z* 102 (100%) resulting from the loss of Br and CO (Table 2 and Fig. 5). In fact, the loss of CO from the acylium ions can be observed in most of the cases with variable intensity, excepting for 4a and 4b.

The amides of type 9 and 10 had the lowest relative abundance of the acylium ions RCO^+^ possibly because of the lack of conjugation to the carbonyl group. In this case, the tropylium cations were observed at *m*/*z* 181 and 135, respectively. The tertiary amide 10, with hydrogen at the γ-carbon and not conjugated, displayed the base peak at *m*/*z* 127, resulting from the McLafferty rearrangement with elimination of safrole as a neutral molecule (Fig. 7).

The 10 different types of amides were further investigated under HRESI-MS conditions to determine similarities in their behavior. First, all 10 amide-types analyzed by HRESI-MS displayed intense ions of protonated molecule [M + H]^+^ (Table 3). Despite of the relative lower basicity of the amide nitrogen, the positive mode is still preferable over the negative one yielding higher intensity of ions (data not shown). As observed for the fragmentation pattern of amides using EI-MS and from previous data,^32^ the aryl acylium ions resulting from the N–CO α-cleavage with the loss of neutral amines, were observed for all amides (Table 3) (Fig. 8). Accordingly, the formation of aryl acylium ion at *m*/*z* 221 from piplartine (1a) and at *m*/*z* 201 from piperine (6a) were observed as reported.^14,16^ Indeed, the acylium ions observed in the EIMS were consistently observed in the ESIMS spectra for all cases (1–10) and such fragmentation is the most important for characterizing the carboxylic acid moieties. The amide 10 showed an aryl acylium ion at *m*/*z* 205 in its HRESI spectrum but also displayed ions resulting from the McLafferty rearrangement similar to what was observed with EI (Fig. 7, Tables 2 and 3).

**Table 3:** Molecular formula, compound exact mass, HRESI [M + H]^+^ and acylium ions (RCO^+^) observed in MS/MS spectra of amides^a^

Amides	Molecular formula	[M + H]^+^ calculated	[M + H]^+^ observed	Error ppm	RCO^+^	Molecular formula
1a	C_17_H_19_NO_5_	318.1336	318.1337	0.31	221.0803	C_12_H_13_O_4_^+^
1b	C_16_H_19_NO_5_	306.1336	306.1345	2.94	221.0859	C_12_H_13_O_4_^+^
1c	C_20_H_31_NO_4_	350.2326	350.2334	2.28	221.0817	C_12_H_13_O_4_^+^
1d	C_17_H_25_NO_4_	308.1856	308.1859	0.97	221.0830	C_12_H_13_O_4_^+^
1e	C_16_H_21_NO_5_	308.1492	308.1496	1.30	221.0822	C_12_H_13_O_4_^+^
1f	C_17_H_23_NO_4_	306.1700	306.1704	1.31	221.0815	C_12_H_13_O_4_^+^
2a	C_19_H_29_NO_3_	320.2220	320.2226	1.87	191.0703	C_11_H_11_O_3_^+^
2b	C_16_H_23_NO_3_	278.1751	278.1758	2.52	191.0702	C_11_H_11_O_3_^+^
2c	C_15_H_19_NO_4_	278.1387	278.1389	0.72	191.0706	C_11_H_11_O_3_^+^
2d	C_16_H_21_NO_3_	276.1594	276.1602	2.90	191.0700	C_11_H_11_O_3_^+^
2e	C_15_H_17_NO_4_	276.1230	276.1235	1.81	191.0702	C_11_H_11_O_3_^+^
3a	C_13_H_14_BrNO_2_	296.0281	296.0282	0.34	208.9588	C_9_H_6_BrO^+^
3b	C_14_H_18_BrNO	296.0645	296.0647	0.68	208.9585	C_9_H_6_BrO^+^
3c	C_17_H_24_BrNO	338.1114	338.1116	0.59	208.9533	C_9_H_6_BrO^+^
4a	C_14_H_15_NO_4_	262.1074	262.1072	−0.76	175.0384	C_10_H_7_O_3_^+^
4b	C_18_H_25_NO_3_	304.1907	304.1902	−1.64	175.0380	C_10_H_7_O_3_^+^
5a	C_17_H_25_NO	260.2008	260.2010	0.77	131.0430	C_9_H_7_O^+^
5b	C_14_H_19_NO	218.1539	218.1538	−0.46	131.0487	C_9_H_7_O^+^
5c	C_13_H_15_NO_2_	218.1175	218.1180	2.29	131.0482	C_9_H_7_O^+^
5d	C_13_H_13_NO_2_	216.1019	216.1015	−1.85	131.0480	C_9_H_7_O^+^
6a	C_17_H_19_NO_3_	286.1438	286.1441	1.05	201.0556	C_12_H_10_O_3_^+^
6b	C_17_H_21_NO_3_	288.1594	288.1596	0.69	201.0535	C_12_H_10_O_3_^+^
7a	C_17_H_23_NO_3_	274.1802	274.1800	−0.73	187.0744	C_12_H_12_O_2_^+^
7b	C_20_H_29_NO_2_	316.2260	316.2258	−0.63	187.0743	C_12_H_12_O_2_^+^
7c	C_17_H_21_NO_2_	272.1645	272.1646	0.37	187.0744	C_12_H_12_O_2_^+^
8a	C_16_H_20_BrNO	322.0801	322.0801	0.00	234.9745	C_11_H_9_BrO^+^
8b	C_16_H_18_BrNO	320.0645	320.0649	1.25	234.9749	C_11_H_9_BrO^+^
9	C_17_H_23_NO_5_	322.1649	322.1649	0.00	223.0969	C_12_H_15_O_4_^+^
10	C_17_H_23_NO_3_	290.1751	290.1752	0.34	205.0845	C_12_H_14_O_3_^+^

a RCO^+^ = [[M + H] − NR_d_R_e_].

**Fig. 3:**
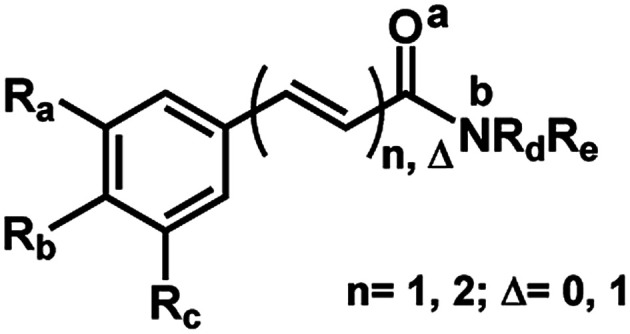
General structures of amides.

In order to determine the preferable protonation sites for each amides in HRESI-MS, and to further elucidate the fragmentation mechanisms, the proton affinity of five amides was calculated for two main binding sites: carbonyl oxygen and amide nitrogen (a and b in Fig. 3, respectively). Five amides (1c, 1d, 1f, 6a and 10) were used as a template because their representative structures and because it was assumed that their differing substituents in the aryl group would not affect significantly the overall results. In general, compounds with proton a bonded to the nitrogen have relatively lower energy, with Δ*P* ranging from 7.6 to 35.7 kJ mol^−1^ (Table 4), supporting the formation of a protonated molecular ion with the proton preferentially bonded to the nitrogen. Thus, the elimination of the amine molecules would facilitate the formation of acylium cations.

**Table 4:** Proton affinity (kJ mol^−1^) of amide (PA)^a^

Amides	PA(a)	PA(b)	Δ*P*
1c	933.66	903.37	30.29
1d	973.89	946.86	27.03
1f	984.40	976.82	7.58
6a	996.12	976.24	19.88
10	980.89	945.19	35.70

a a and b: bonding sites for hydrogens (Fig. 3).

The hydrogen bonding energy to amide nitrogen and carbonyl were calculated with Spartan software. The application of DFT at the B3LYP level, generated a set of bond energies that can be compared. The calculated bond energy values calculated by DFT-B3LYP of all the amides show that the *E*_b_ (N–C bond energy with proton attached to amide nitrogen) energies were significantly lower than the *E*_a_ (N–C bond energy with proton attached to the carboxyl oxygen) (Table 6) energies or not protonated amides (*E*). Therefore, the fragmentation of the N–C bond is preferable in order to generate the acylium ions when the proton is attached to amide nitrogen as it would be mechanistically expected.

**Fig. 4:**
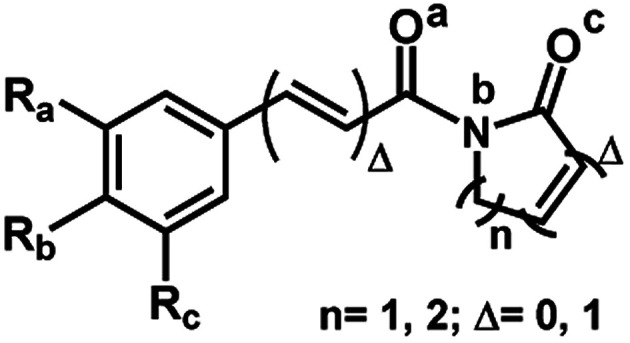
General structures of imides.

Similar profiles of energies were observed for imide derivatives (1a, 1b, 2e, 5d and 9) (Fig. 4 and Table 5). The PA (Proton Affinity) values were lower with the hydrogens bonded to the nitrogen than to the carbonyl oxygen at position a or c (Fig. 4 and Table 5).

**Fig. 5:**
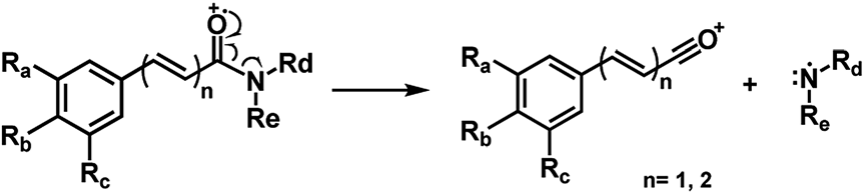
Main fragmentation pattern of the amides in EI-MS.

**Fig. 6:**
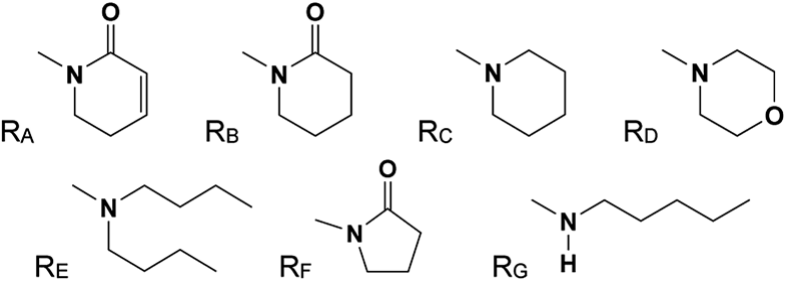
Amine (R_C_, R_D_, R_E_, R_G_) and lactame (R_A_, R_B_, R_F_) moieties.

**Fig. 7:**
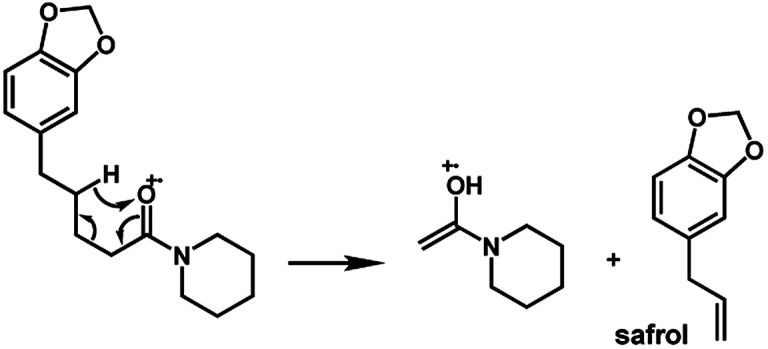
McLafferty rearrangement for amide 10 under EI conditions.

**Fig. 8:**
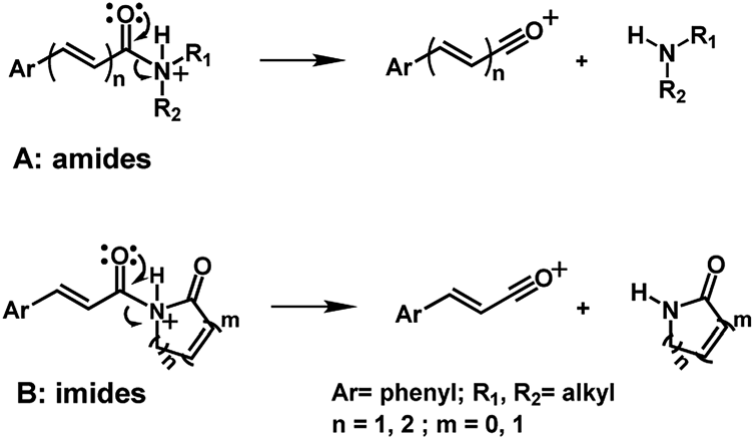
Main fragmentation pathway for amides (A) and imides (B) in HRESI-MS.

**Table 5:** Proton affinity (kJ mol^−1^) of imides (PA)^a^

Amide	PA(a)	PA(b)	PA(c)
1a	945.75	898.82	927.77
1b	951.73	887.15	899.90
2e	981.27	885.84	899.73
5d	951.08	891.88	917.21
9	941.94	889.96	946.81

a a, b and c: bonding sites for hydrogens (Fig. 4).

**Table 6:** Bond energy (kJ mol^−1^) of amides with proton bonded at position a and b of the N–C bond (*E*, *E*_a_, *E*_b_) calculated with Spartan 16 software^a^

Amides	*E*	*E*_a_	*E*_b_
1c	1695.86	211.93	167.08
1d	948.43	222.97	179.04
1e	890.08	210.00	180.59
1f	912.47	228.24	198.91
3a	846.79	125.30	108.94
3b	902.95	159.94	109.53
3c	1528.89	152.62	121.72
4a	801.33	121.38	99.34
4b	1490.65	133.37	101.17
5a	1522.02	151.48	100.01
5b	891.07	157.18	94.95
5c	833.14	147.35	116.79
6a	803.07	120.94	104.79
6b	838.06	125.59	96.50
7a	838.07	124.79	94.25
7b	1482.96	132.95	105.32
7c	801.67	129.69	102.17
8a	869.14	143.18	91.71
8b	822.19	147.12	108.35
10	930.22	208.93	148.45

a a and b: bonding sites for hydrogens (Fig. 3).

We also calculated the bond energies at position 1 of N–C various of imides derivatives. The values of the bond energies calculated by DFT-B3LYP for all of the imides show that the *E*_b1_ (N–C bond energy with proton attached to amide nitrogen) energies were notably lower than *E*_a1_ and *E*_c1_ (Table 7). Specifically, in the case of compound 9, which lacks conjugation between the carbonyl and the phenyl group, the bond energy difference is the smallest (Table 7). Thus, the fragmentation of the N–C is similarly preferred when the proton is attached to imide nitrogen.

**Table 7:** Bond energy (kJ mol^−1^) of imides and proton bonded at a, b and c and of the N–C bonded at position 1 (*E*_1_, *E*_a1_, *E*_b1_, *E*_c1_) calculated by Spartan 16 software^a^

position	1a	1b	2e	5d	9
*E*_1_	764.92	774.95	678.95	731.28	770.14
*E*_a1_	148.35	168.29	86.81	108.23	99.55
*E*_b1_	115.22	97.53	11.76	38.21	84.98
*E*_c1_	139.98	124.70	39.36	67.22	112.60

a a, b and c: bonding sites for hydrogens (Fig. 4).

## 4. Conclusions

In this work we compared the fragmentation pattern of two natural piperamides (piplartine and piperine) and several synthetic derivatives that were analysed under HRESI-MS and EI-MS conditions. Piplartine and piperine (α,β-unsaturated amides) have shown a N–CO α-cleavage, a characteristic fragmentation observed as a common pattern in all natural and synthetic amides. Fragmentation mechanisms were proposed based on computational analysis using DFT-B3LYP to calculate proton affinities and bond energies. The computational methods supported the fragmentation mechanism proposed in the HRESI-MS experiment involving the protonation of piperamides and derivatives preferentially in the amide nitrogen.

## Conflicts of interest

There are no conflicts to declare.

## Supplementary Material

RA-008-C7RA00408G-s001

## References

[cit1] Wijeratne E. M. K., Bandara B. M. R., Gunatilaka A. A. L., Tezuka Y., Kikuchi T. (1992). J. Nat. Prod..

[cit2] Sun J., Huo H.-X., Zhang J., Huang Z., Zheng J., Zhang Q., Zhao Y.-F., Li J., Tu P.-F. (2015). Biochem. Syst. Ecol..

[cit3] Ali M. S., Ahmed G., Pervez M. K. (2012). J. Chem. Soc. Pak..

[cit4] Okwute S. K., Egharevba H. O. (2013). Int. J. Chem..

[cit5] Guzman I. , Bosland P. W. and O'Connell M. A., in Recent Advances in Phytochemistry 41: The Biological Activity of Phytochemicals, ed. D. R. Gang, Springer, New York, 2011

[cit6] Dyer L. A. , Richards J. and Dodson C. D., in Piper. A model genus for studies of evolution, chemical ecology, and trophic interactions, ed. L. A. Dyer and A. N. Palmer, Kluwer Academic Publishers, Boston, 2004, pp. 117–139

[cit7] Bezerra D. P., Pessoa C., Moraes M. O. d., Alencar N. M. N. d., Mesquita R. O., Lima M. W., Alves A. P. N. N., Pessoa O. D. L., Chaves J. H., Silveira E. R., Costa-Lotufo L. V. (2008). J. Appl. Toxicol..

[cit8] Bezerra D. P., Castro F. O. d., Alves A. P. N. N., Pessoa C., Moraes M. O. d., Silveira E. R., Lima M. A. S., Elmiro F. J. M., Alencar N. M. N. d., Mesquita R. O., Lima M. W., Costa-Lotufo L. V. (2008). J. Appl. Toxicol..

[cit9] Gutierrez R. M., Gonzalez A. M., Hoyo-Vadillo C. (2011). Mini-Rev. Med. Chem..

[cit10] Marques J. V., Oliveira A. d., Raggi L., Young M. C. M., Kato M. J. (2010). J. Braz. Chem. Soc..

[cit11] da Silva R. V., Navickiene H. M. D., Kato M. J., Bolzani V. S., Meda C. I., Young M. C. M., Furlan M. (2002). Phytochemistry.

[cit12] Navickiene H. M. D., Alécio A. C., Kato M. J., Bolzani V. S., Young M. C. M., Cavalheiro A. J., Furlan M. (2000). Phytochemistry.

[cit13] Achenbach H., Fietz W., Wörth J., Waibel R., Portecop J. (1986). Planta Med..

[cit14] Bajad S., Coumar M., Khajuria R., Suri O. P., Bedi K. L. (2003). Eur. J. Pharm. Sci..

[cit15] da Silva-Junior E. A., Paludo C. R., Gouvea D. R., Kato M. J., Furtado N. A. J. C., Lopes N. P., Vessecchi R., Pupo M. T. (2017). J. Mass Spectrom..

[cit16] Schaab E. H., Crotti A. E. M., Iamamoto Y., Kato M. J., Lotufo L. V. C., Lopes N. P. (2010). Biol. Pharm. Bull..

[cit17] Bezerra D. P., Castro F. O., Alves A. P. N. N., Pessoa C., Moraes M. O., Silveira E. R., Lima M. A. S., Elmiro F. J. M., Costa-Lotufo L. V. (2006). Braz. J. Med. Biol. Res..

[cit18] Chang-Yih D., Yang-Chang W., Shang-Kwei W. (1990). Phytochemistry.

[cit19] Parmar V. S., Jain S. C., Bisht K. S., Jain R., Taneja P., Jha A., Tyagi O. D., Prasad A. K., Wengel J., Olsen C. E., Boll P. M. (1997). Phytochemistry.

[cit20] De Araujo-Junior J. o. X., Da-Cunha E. V. L., Chaves M. C. l. D. O., Gray A. I. (1997). Phytochemistry.

[cit21] Stein M., Breit B. (2013). Angew. Chem., Int. Ed..

[cit22] Becke A. D. (1988). Phys. Rev. A.

[cit23] Vereecken L., Pierloot K., Peeters J. (1998). J. Chem. Phys..

[cit24] Mezzache S., Pepe C., Karoyan P., Fournier F., Tabet J. C. (2005). Rapid Commun. Mass Spectrom..

[cit25] Pepe C., Rochut S., Paumard J. P., Tabet J. C. (2004). Rapid Commun. Mass Spectrom..

[cit26] Mezzache S., Afonso C., Pepe C., Karoyan P., Fournier F., Tabet J. C. (2003). Rapid Commun. Mass Spectrom..

[cit27] Tirado-Rives J., Jorgensen W. L. (2008). J. Chem. Theory Comput..

[cit28] Halgren T. A. (1996). J. Comput. Chem..

[cit29] Stewart J. J. P. (2013). J. Mol. Model..

[cit30] Barker J. , Mass spectrometry, John Wiley & Sons Ltd, New York, 1999

[cit31] Gross J. H. , Mass spectrometry, a textbook, Springer, Heidelberg, 2nd edn, 2011

[cit32] Senda N., Wakayama H., Fujita T., Bando T., Shizukuishi K., Yamaoka H., Nakayama M. (1994). J. Mass Spectrom. Soc. Jpn..

