# Supplementary material for: Fragmentation pattern of amides by EI and HRESI: study of protonation sites using DFT-3LYP data
Source: RSC Adv. 2018 Jun 12;8(38):21407–13. doi: 10.1039/c7ra00408g (PMC9080946; doi:10.1039/c7ra00408g)
Supplement: RA-008-C7RA00408G-s001 [file RA-008-C7RA00408G-s001.pdf]

Electronic Supplementary Material (ESI) for RSC Advances.
This journal is © The Royal Society of Chemistry 2018

Supporting Information

Spectral data of amides (**1a**-**10**)

Fig S1 EI spectrum of **1a** (1-[(2*E*)-3-(3,4,5-trimethoxyphenyl)prop-2-enoyl]-1,2,5,6-tetrahydropyridin-2-one)

Fig S2 HRESI spectrum of **1a** (1-[(2*E*)-3-(3,4,5-trimethoxyphenyl)prop-2-enoyl]-1,2,5,6-tetrahydropyridin-2-one)

Fig S3 EI spectrum of **1b** (1-[(2*E*)-3-(3,4,5-trimethoxyphenyl)prop-2-enoyl]pyrrolidin-2-one

Fig S4 HRESI spectrum of **1b** (1-[(2*E*)-3-(3,4,5-trimethoxyphenyl)prop-2-enoyl]pyrrolidin-2-one

Fig S5 EI spectrum of **1c** (2E)-*N,N*-dibutyl-3-(3,4,5-trimethoxyphenyl)prop-2-enamide

Fig S6 HRESI spectrum of **1c** (2E)-*N,N*-dibutyl-3-(3,4,5-trimethoxyphenyl)prop-2-enamide

Fig S7 EI spectrum of **1d** (2*E*)-*N*-pentyl-3-(3,4,5-trimethoxyphenyl)prop-2-enamide

Fig S8 HRESI spectrum of **1d** (2*E*)-*N*-pentyl-3-(3,4,5-trimethoxyphenyl)prop-2-enamide

Fig S9 EI spectrum of **1e** (2*E*)-1-(morpholin-4-yl)-3-(3,4,5-trimethoxyphenyl)prop-2-en-1-one

Fig S10 HRESI spectrum of **1e** (2*E*)-1-(morpholin-4-yl)-3-(3,4,5-trimethoxyphenyl)prop-2-en-1-one

Fig S11 EI spectrum of **1f** (2*E*)-1-(piperidin-1-yl)-3-(3,4,5-trimethoxyphenyl)prop-2-en-1-one

Fig S12 HRESI spectrum of **1f** (2*E*)-1-(piperidin-1-yl)-3-(3,4,5-trimethoxyphenyl)prop-2-en-1-one

Fig S13 EI spectrum of **2a** (2*E*)-*N,N*-dibutyl-3-(3,4-dimethoxyphenyl)prop-2-enamide

Fig S14 HRESI spectrum of **2a** (2*E*)-*N,N*-dibutyl-3-(3,4-dimethoxyphenyl)prop-2-enamide

Fig S15 EI spectrum of **2b** (2*E*)-3-(3,4-dimethoxyphenyl)-*N*-pentylprop-2-enamide

Fig S16 HRESI spectrum of **2b** (2*E*)-3-(3,4-dimethoxyphenyl)-*N*-pentylprop-2-enamide

Fig S17 EI spectrum of **2c** (2*E*)-3-(3,4-dimethoxyphenyl)-1-(morpholin-4-yl)prop-2-en-1-one

Fig S18 HRESI spectrum of **2c** (2*E*)-3-(3,4-dimethoxyphenyl)-1-(morpholin-4-yl)prop-2-en-1-one

Fig S19 EI spectrum of **2d** (2*E*)-3-(3,4-dimethoxyphenyl)-1-(piperidin-1-yl)prop-2-en-1-one

Fig S20 HRESI spectrum of **2d** (2*E*)-3-(3,4-dimethoxyphenyl)-1-(piperidin-1-yl)prop-2-en-1-one

Fig S21 EI spectrum of **2e** 1-[(2*E*)-3-(3,4-dimethoxyphenyl)prop-2-enoyl]pyrrolidin-2-one

Fig S22 HRESI spectrum of **2e** 1-[(2*E*)-3-(3,4-dimethoxyphenyl)prop-2-enoyl]pyrrolidin-2-one

Fig S23 EI spectrum of **3a** (2*E*)-3-(4-bromophenyl)-1-(morpholin-4-yl)prop-2-en-1-one

Fig S24 HRESI spectrum of **3a** (2*E*)-3-(4-bromophenyl)-1-(morpholin-4-yl)prop-2-en-1-one

Fig S25 EI spectrum of **3b** (2*E*)-3-(4-bromophenyl)-*N*-pentylprop-2-enamide

Fig S26 HRESI spectrum of **3b** (2*E*)-3-(4-Bromophenyl)-*N*-pentylprop-2-enamide

Fig S27 EI spectrum of **3c** (2*E*)-3-(4-bromophenyl)-*N,N*-dibutylprop-2-enamide

Fig S28 HRESI spectrum of **3c** (2*E*)-3-(4-bromophenyl)-*N,N*-dibutylprop-2-enamide

Fig S29 EI spectrum of **4a** (2*E*)-3-(2*H*-1,3-benzodioxol-5-yl)-1-(morpholin-4-yl)prop-2-en-1-one

Fig S30 HRESI spectrum of **4a** (2*E*)-3-(2*H*-1,3-Benzodioxol-5-yl)-1-(morpholin-4-yl)prop-2-en-1-one

Fig S31 EI spectrum of **4b** (2*E*)-3-(2H-1,3-benzodioxol-5-yl)-*N,N*-dibutylprop-2-enamide

Fig S32 HRESI spectrum of **4b** (2*E*)-3-(2H-1,3-benzodioxol-5-yl)-*N,N*-dibutylprop-2-enamide

Fig S33 EI spectrum of **5a** (2*E*)-*N,N*-dibutyl-3-phenylprop-2-enamide

Fig S34 HRESI spectrum of **5a** (2*E*)-*N,N*-dibutyl-3-phenylprop-2-enamide

Fig S35 EI spectrum of **5b** (2*E*)-*N*-pentyl-3-phenylprop-2-enamide

Fig S36 HRESI spectrum of **5b** (2*E*)-*N*-pentyl-3-phenylprop-2-enamide

Fig S37 EI spectrum of **5c** (2*E*)-1-(morpholin-4-yl)-3-phenylprop-2-en-1-one

Fig S38 HRESI spectrum of **5c** (2*E*)-1-(morpholin-4-yl)-3-phenylprop-2-en-1-one

Fig S39 EI spectrum of **5d** 1-[(2*E*)-3-phenylprop-2-enoyl]pyrrolidin-2-one

Fig S40 HRESI spectrum of **5d** 1-[(2*E*)-3-phenylprop-2-enoyl]pyrrolidin-2-one

Fig S41 EI spectrum of **6a** (2*E*,4*E*)-5-(2*H*-1,3-benzodioxol-5-yl)-1-(piperidin-1-yl)penta-2,4-dien-1-one

Fig S42 HRESI spectrum of **6a** (2*E*,4*E*)-5-(2*H*-1,3-benzodioxol-5-yl)-1-(piperidin-1-yl)penta-2,4-dien-1-one

Fig S43 EI spectrum of **6b** (2*E*,4*E*)-5-(2*H*-1,3-benzodioxol-5-yl)-*N*-pentylpenta-2,4-dienamide

Fig S44 HRESI spectrum of **6b** (2*E*,4*E*)-5-(2*H*-1,3-benzodioxol-5-yl)-*N*-pentylpenta-2,4-dienamide

Fig S45 EI spectrum of **7a** (2*E*,4*E*)-5-(4-methoxyphenyl)-*N*-pentylpenta-2,4-dienamide

Fig S46 HRESI spectrum of **7a** (2*E*,4*E*)-5-(4-methoxyphenyl)-*N*-pentylpenta-2,4-dienamide

Fig S47 EI spectrum of **7b** (2*E*,4*E*)-*N,N*-dibutyl-5-(4-methoxyphenyl)penta-2,4-dienamide

Fig S48 HRESI spectrum of **7b** (2*E*,4*E*)-*N,N*-dibutyl-5-(4-methoxyphenyl)penta-2,4-dienamide

Fig S49 EI spectrum of **7c** (2*E*,4*E*)-5-(4-methoxyphenyl)-1-(piperidin-1-yl)penta-2,4-dien-1-one

Fig S50 HRESI spectrum of **7c** (2*E*,4*E*)-5-(4-methoxyphenyl)-1-(piperidin-1-yl)penta-2,4-dien-1-one

Fig S51 EI spectrum of **8a** (2*E*,4*E*)-5-(4-bromophenyl)-*N*-pentylpenta-2,4-dienamide

Fig S52 HRESI spectrum of **8a** (2*E*,4*E*)-5-(4-bromophenyl)-*N*-pentylpenta-2,4-dienamide

Fig S53 EI spectrum of **8b** (2*E*,4*E*)-5-(4-bromophenyl)-1-(piperidin-1-yl)penta-2,4-dien-1-one

Fig S54 HRESI spectrum of **8b** (2*E*,4*E*)-5-(4-bromophenyl)-1-(piperidin-1-yl)penta-2,4-dien-1-one

Fig S55 EI spectrum of **9** 1-[3-(3,4,5-trimethoxyphenyl)propanoyl]piperidin-2-one

Fig S56 HRESI spectrum of **9** 1-[3-(3,4,5-trimethoxyphenyl)propanoyl]piperidin-2-one

Fig S57 EI spectrum of **10** (2*E*,4*E*)-5-(2*H*-1,3-benzodioxol-5-yl)-1-(piperidin-1-yl)penta-2,4-dien-1-one

Fig S58 HRESI spectrum of **10** (2*E*,4*E*)-5-(2*H*-1,3-benzodioxol-5-yl)-1-(piperidin-1-yl)penta-2,4-dien-1-one

Spectral data of amides (1a-10)

1a: 1-[(2*E*)-3-(3,4,5-trimethoxyphenyl)prop-2-enoyl]-1,2,5,6-tetrahydropyridin-2-one

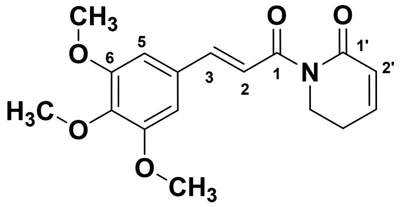

^{1}H NMR (300 MHz; $CDCl_3$): δ 7.68 (d; *J*=15.6 Hz; 1H; H-2); 7.43 (d; *J*=15.6 Hz; 1H; H-3); 6.95 (m; 1H; H-2'); 6.81 (s; 2H; H-5; H-9); 6.04 (dt; *J*=9.9 and 1.8 Hz; 1H; H-3'); 4.05 (t; *J*=6.5; 2H; H-5'); 3.89 (s; 6H; OMe-6 and 8) 3.88 (s; 3H; OMe-7); 2.50 (m; 2H, H-4'). ^{13}C NMR (75 MHz; $CDCl_3$): δ 168.70 (C-1); 165.76 (C-1'); 153.30 (C-6; C-8); 145.50 (C-3'); 143.70 (C-4); 139.90 (C-7); 130.60 (C-3); 125.70 (C-2'); 121.00 (C-2); 105.40 (C-5; C-9); 60.89 (OMe-7); 56.11 (OMe-6 and 8); 41.62 (C-5'); 24.70 (C-4'). EI-MS (*m/z*) (%): 317 (90; M^+); 274 (32); 221 (100); 190 (32). HRESI-MS: $[M+H]^+$ (*m/z*) calculated: 318.1336; found 318.1337.

1b: **1-[(2*E*)-3-(3,4,5-trimethoxyphenyl)prop-2-enoyl]pyrrolidin-2-one**

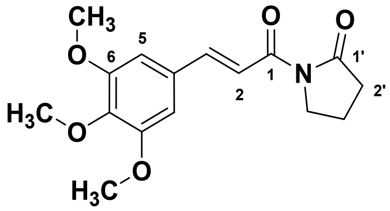

^{1}H NMR (300 MHz; $CDCl_3$): δ 7.85 (d; *J*=15.6 Hz; 1H; H-2); 7.75 (d; *J*=15.6 Hz; 1H; H-3); 6.84 (s; 2H; H-5; H-9); 3.93 (m; 2H; H-4'); 3.90 (s; 6H; OMe-6 and 8); 3.89 (s; 3H; OMe-7); 2.66 (t; *J*=9.1 Hz; 2H, H-2'); 2.09 (m; 2H; H-3'). ^{13}C NMR (75 MHz; $CDCl_3$): δ 164.52 (C-1); 153.25 (C-6, C-8); 141.64 (C-3); 139.40 (C-7); 130.80 (C-4); 117.98 (C-2); 104.95 (C-5, C-9); 60.82 (OMe-7); 56.07 (OMe-6 and 8); 46.51 (C-1'); 45.96 (C-4'); 26.02 (C-2'); 24.23 (C-3'). EI-MS (*m/z*) (%): 305 (100; M^+); 221 (55); 205 (40). HRESI-MS: $[M+H]^+$ (*m/z*) calculated: 306.1336 ; found: 306.1345.

1c: **(2E)-*N,N*-dibutyl-3-(3,4,5-trimethoxyphenyl)prop-2-enamide**

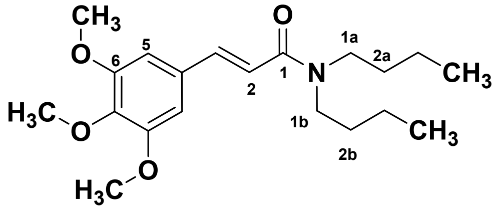

^{1}H NMR (200 MHz; $CDCl_3$): δ 7.61 (d; *J*= 15.2 Hz; 1H; H-3); 6.73 (s; 2H; H-5, H-9); 6.72 (d; *J*= 15.2 Hz; 1H; H-2); 3.89 (s; 6H; OMe-6 and 8); 3.87 (s; 3H; OMe-7); 3.42 (m; 4H; H-1); 1.59 (m; 4H; H-2a; H-2b); 1.36 (m; 4H; H3a; H3b); 0.96 (m; 6H; H4a; H4b). ^{13}C NMR (50 MHz; $CDCl_3$): δ 165.78 (C-1); 153.22 (C-6; C-8); 141.98 (C-3); 139.33 (C-7); 131.01 (C-4); 117.11 (C-2); 104.82 (C-5, C-9); 60.78 (OMe-7); 56.00 (OMe-6 and 8); 47.68 (C-1a); 46.49 (C-1b); 31.72 (C-2a); 29.94 (C-2b); 20.14 (C-3a); 19.87 (C-3b); 13.74 (C-4a); 13.62 (C-4b). EI-MS (*m/z*) (%): 349 (37, M^+); 306 (15); 222 (63); 221 (100). HRESI-MS: $[M+H]^+$ (*m/z*) calculated: 350.2326; found: 350.2334.

1d: **(2*E*)-*N*-pentyl-3-(3,4,5-trimethoxyphenyl)prop-2-enamide**

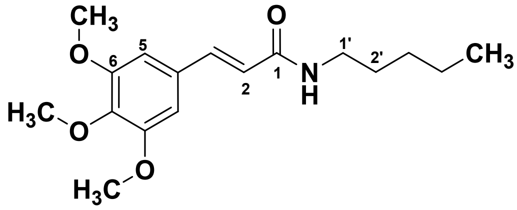

^{1}H NMR (200 MHz; $CDCl_3$): δ 7.54 (d; *J*= 15.6 Hz; 1H; H-3); 6.73 (s; 2H; H5; H-9); 6.32 (d; *J*= 15.6 Hz; 1H; H-2); 3.88 (s; 6H; OMe-6 and 8); 3.87 (s; 3H; OMe-7); 3.39 (t; *J*= 6.7 Hz; 2H, H-1'); 1.58 (m; 2H; H-2'); 1,35 (m; 4H; H3'; H-4'); 0.91 (t; *J*= 7.4 Hz; 3H; H-5'). ^{13}C NMR (50 MHz; $CDCl_3$): δ 165.72 (C-1); 153.36 (C-6; C-8); 140.73 (C-3); 139.50 (C-7); 130.45 (C-4); 120.15 (C-2); 104.88 (C-5, C-9); 60.91 (OMe-7); 56.10 (OMe-6 and 8); 39.75 (C-1'); 29.35 (C-2'); 29.08 (C-3'); 22.35 (C-4'); 13.95 (C-5').EI-MS (*m/z*) (%): 307 (85; M^+); 236 (45); 222 (100); 221 (95); 190 (30); 181 (55). HRESI-MS: $[M+H]^+$ (*m/z*) calculated: 308.1856; found: 308.1859.

1e: **(2*E*)-1-(Morpholin-4-yl)-3-(3,4,5-trimethoxyphenyl)prop-2-en-1-one**

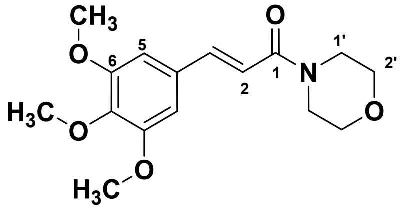

^{1}H NMR (200 MHz; $CDCl_3$): δ 7.62 (d; *J*= 15.4 Hz; 1H; H-3); 6.74 (s; 2H; H5; H-9); 6.72 (d; *J*= 15.4 Hz; 1H; H-2); 3.90 (s; 6H; OMe 6 and 8); 3.88 (s; 3H; OMe-7); 3.73 (s; 8H; H-1'; H-2'; H-4'; H-5'). ^{13}C NMR (50 MHz; $CDCl_3$): δ 165.47 (C-1); 153.36 (C-6; C-8); 143.26 (C-3); 139.67 (C-7); 130.61 (C-4); 115.67 (C-2); 105.00 (C-5; C-9); 66,80 (C-2'; C-4');

60.89 (OMe-7), 56.15 (OMe-6 and 8); 45,56 (C-1'); 42,36 (C-5'). EI-MS (*m/z*) (%): 307 (50; M^+); 222 (60); 221 (100); 190 (25). HRESI-MS: $[M+H]^+$ (*m/z*) calculated: 308.1492; found: 308.1496.

1f: **(2*E*)-1-(Piperidin-1-yl)-3-(3,4,5-trimethoxyphenyl)prop-2-en-1-one**

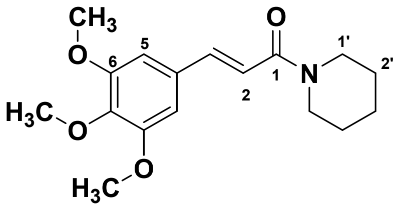

^{1}H NMR (200 MHz; $CDCl_3$): δ 7.56 (d; *J*=15.4 Hz; 1H; H-3); 6.79 (d; *J*=15.4 Hz; 1H; H-2); 6.74 (s; 2H; H-5; H-9); 3.90 (s; 6H; OMe-6 and 8); 3.87 (s; 3H; OMe-7); 3.63 (m; 4H; H-1'; H-5'); 1.88 (m; 2H; H-3'); 1.66 (m; 4H; H-2'; H4'). ^{13}C NMR (50 MHz; $CDCl_3$): δ 165.24 (C1); 153.34 (C6, C8); 142.19 (C3); 139.39 (C7); 131.05 (C4); 116.95 (C2); 104.89 (C5, C9); 60.90 (OMe-7); 56.17(OMe-6 and 8); 46.98 (C1'); 43.34 (C5'); 26.71 (C2'); 25.59 (C4'); 24.60 (C3'). EI-MS (*m/z*) (%): 305 (60; M^+); 222 (100); 221 (65); 194 (25); 191 (35); 190 (25); 179 (28); 84 (69). HRESI-MS: $[M+H]^+$ (*m/z*) calculated: 306.1700; found: 306.1704.

2a: **(2*E*)-*N,N*-Dibutyl-3-(3,4-dimethoxyphenyl)prop-2-enamide**

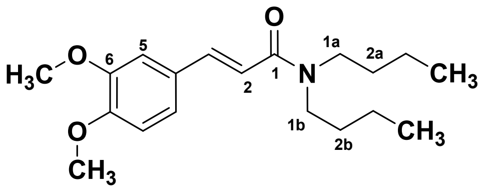

^{1}H NMR (200 MHz; $CDCl_3$): δ 7.64 (d; *J*=15.3 Hz; 1H; H-3); 7.14-7.09 (dd; *J*= 8.4 and 2.0 Hz; 1H, H-9); 7.02 (d; *J*= 2.0 Hz; 1H; H-5); 6.87 (d; *J*= 8.4 Hz; 1H; H-8) 6.70 (d; *J*=15.3 Hz; 1H; H-2); 3.91 (s; 6H; OMe-6 and 7); 3.47-3.36 (m; 4H; H-1a; H-1b); 1.68-1.54 (m; 4H; H-2a; H-2b); 1.44-1.29 (m; 4H; H-3a; H-3b); 1.02-0.91 (m; 6H; H4a; H4b).^{13}C NMR (50 MHz; $CDCl_3$): δ 166.02 (C-1); 150.21 (C-6); 148.91 (C-7); 141.88 (C-3); 128.42 (C-4); 121.28 (C-9); 115.60 (C-2); 110.99 (C-8); 109.99 (C-5); 55.77 (OMe-6); 55.72 (OMe-7); 47.68 (C-1a); 46.48 (C-1b); 31.74 (C-2a); 29.96 (C-2b); 20.33 (C-3a); 19.90 (C-3b); 13.73 (C-4a); 13.65 (C-4b). EI-MS (*m/z*) (%): 319 (20; M^+); 191 (100). HRESI-MS: $M+H]^+$ (*m/z*) calculated: 320.2220; found: 320.2226.

2b: **(2*E*)-3-(3,4-Dimethoxyphenyl)-*N*-pentylprop-2-enamide**

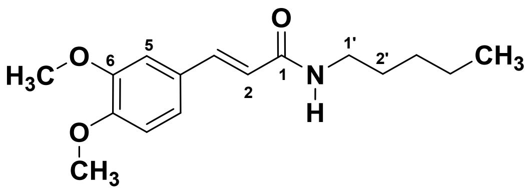

^{1}H NMR (200 MHz; $CDCl_3$): δ 7.56 (d; *J*=15.5 Hz; 1H; H-3); 7.11-7.06 (dd; *J*= 8.4and 1.8 Hz; 1H; H-9); 7.02 (d; *J*= 1.8 Hz; 1H; H-5) 6.85 (d; *J*= 8.4 Hz; 1H; H-8); 6.26 (d; *J*= 15.5 Hz; 1H; H-2); 3.91 (s; 6H; OMe-6 and 7); 3.38 (m; 2H; H-1'); 1.61-1.54 (m; 2H; H-2'). 1.38-1.32 (m; 4H; H-3', H-4'). 0.91 (t; *J*= 7.2 Hz; 3H; H-5'). ^{13}C NMR (50 MHz; $CDCl_3$): δ 166.03 (C-1); 150.51 (C-6); 149.11 (C-7); 140.66 (C-3); 127.87 (C-4); 121.85 (C-9); 118.68 (C-2); 111.08 (C-8); 109.66 (C-5); 55.94 (OMe-6); 55.86 (OMe-7); 39.73 (C-1'); 29.41 (C-2'); 29.10 (C-3'); 22.37 (C-4'); 13.98 (C-5'). EI-MS (*m/z*) (%): 277 (43; M^+); 206 (35); 192 (40); 191 (100); 151 (45). HRESI-MS: $[M+H]^+$ (*m/z*) calculated: 278.1751; found: 278.1758.

2c: **(2*E*)-3-(3,4-Dimethoxyphenyl)-1-(morpholin-4-yl)prop-2-en-1-one**

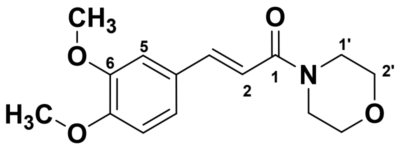

^{1}H NMR (200 MHz; $CDCl_3$): δ 7.66 (d; *J*= 15.4 Hz;1H; H-3);7.14 (dd; *J*= 8.4 and 2.0 Hz; 1H; H-8); 7.04 (d; *J*= 2.0 Hz; 1H; H-5); 6.87 (d; *J*= 8.4 Hz; 1H; H-9); 6.71 (d; *J*=15.4 Hz; 1H; H-2); 3.93 (s; 3H; OMe-6); 3.92 (s; 3H; OMe-7); 3.73

(s; 8H; H-1'; H-2', H-4'; H-5'). ^{13}C NMR (50 MHz; $CDCl_3$): δ 165.77 (C-1); 150.63 (C-6); 149.10 (C-7); 143.20 (C-3); 128.07 (C-4); 121.86 (C-9); 114.16 (C-2); 111.07 (C-8); 109.86 (C-5); 66.83 (C-2'; C-4'); 55.91 (OMe-6 and 7); 45.61 (C-1'); 42.69 (C-5'). EI-MS (*m/z*) (%): 277 (31; M^+); 192 (23); 191 (100). HRESI-MS: $[M+H]^+$ (*m/z*) calculated: 278.1387; found: 278.1389.

2d: **(2*E*)-3-(3,4-Dimethoxyphenyl)-1-(piperidin-1-yl)prop-2-en-1-one**

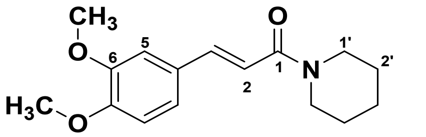

^1H NMR (200 MHz; $CDCl_3$): δ 7.60 (d; *J*=15.4 Hz; 1H; H-3); 7.14-7.08 (dd; *J*= 8.3 and 2.0 Hz; 1H; H-8); 7.03 (d; *J*= 2.0 Hz; 1H; H-5); 6.86 (d; *J*= 8.3 Hz; 1H; H-9); 6.76 (d; *J*=15.4 Hz; 1H; H-2); 3.93 (s; 3H; OMe-6); 3.91 (s; 3H; OMe-7); 3.64 (m; 4H; H-1', H-5'); 1.64 (m; 6H; H2', H3', H4'). ^{13}C NMR (50 MHz; $CDCl_3$): δ 165.38 (C-1); 150.20 (C-6); 148.91 (C-7); 141.99 (C-3); 128.32 (C-4); 121.48 (C-9); 115.30 (C-2); 110.92 (C-8); 109.71 (C-5); 55.78 (OMe-6 and 7); 46.85 (C-1'); 43.21 (C-5'); 26.60 (C-2'); 25.52 (C-4'); 24.50 (C-3'). EI-MS (*m/z*) (%): 275 (55; M^+).192 (35); 191 (100); 84 (42). HRESI-MS: $[M+H]^+$ (*m/z*) calculated: 276.1594; found: 276.1602.

2e: 1-[(2*E*)-3-(3,4-Dimethoxyphenyl)prop-2-enoyl]pyrrolidin-2-one

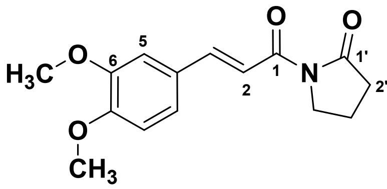

^1H NMR (300 MHz; $CDCl_3$): δ 7.83 (d; *J*= 15.6 Hz; 1H; H-2); 7.78 (d; *J*= 15.6 Hz;1H; H-3); 7.19 (dd; *J*= 8.1 and 1.8 Hz; 1H; H9); 7.14 (d; *J*= 1.8 Hz; 1H; H5); 6.87 (d; *J*= 8.1 Hz; 1H; H-9); 3.95-3.90 (m; 2H; H-4'); 3.93 (s; 3H; OMe-6); 3.92 (s; 3H; OMe-7); 2.65 (t; *J*= 7.8 Hz; 2H, H-2'); 2.12-2.02 (m; 2H; H-3'). ^{13}C NMR (75 MHz; $CDCl_3$): δ 175.75 (C-1'); 166.48 (C-1); 151.28 (C-6); 149.17 (C-7); 145.64 (C-3); 127.99 (C-4); 123.19 (C-9); 116.73 (C-2); 110.99 (C-5); 110.08 (C-8); 55.99 (OMe-6); 55.93 (OMe-7); 45.94 (C-4'); 34.08 (C-2'); 17.23 (C-3'). EI-MS (*m/z*) (%): 275 (51; M^+); 191 (100); HRESI-MS: $[M+H]^+$ (*m/z*) calculated: 276.1230; found: 276.1235.

3a: (2*E*)-3-(4-Bromophenyl)-1-(morpholin-4-yl)prop-2-en-1-one

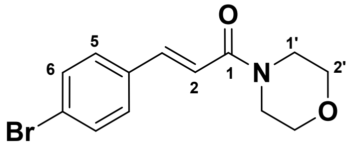

^1H NMR (300 MHz; $CDCl_3$): δ 7.63 (d; *J*= 15.3; 1H, H-3); 7.60-7.48 (dt; *J*= 8.4 and 1.8 Hz; 2H; H-6, H-8); 7.40-7.36 (dt; *J*=8.4 and 1.8 Hz; 2H; H-5, H-9); 6.84 (d; *J*=15.3 Hz; 1H; H-2); 3.72 (s; 8H; H-1', H-2', H-4', H-5').^{13}C NMR (75 MHz; $CDCl_3$): δ 165.16 (C-1); 141.80 (C-3); 133.98 (C-4); 131.97 (C-6, C-8); 129.12 (C-5, C-9); 123.79 (C-7); 117.15 (C2); 66.77 (C2', C4'); 46.19 (C1'); 42.44 (C5'). EI-MS (*m/z*) (%): 297 (21); 295 (21; M^+); 211 (70); 209 (72); 126 (27); 102 (100); 86 (52); 56 (34). HRESI-MS: $[M+H]^+$ (*m/z*) calculated: 296.0281; found: 296.0282.

3b: (2*E*)-3-(4-Bromophenyl)-*N*-pentylprop-2-enamide

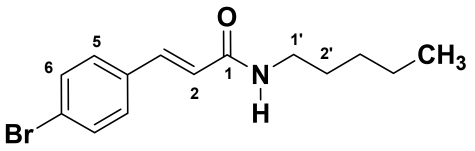

^1H NMR (200 MHz; $CDCl_3$): δ 7.56 (d; *J*= 15.6 Hz; 1H; H-3); 7.51-7.45 (dt; *J*= 8.6 and 2.0 Hz; 2H; H-6; H-8); 7.39-7.32 (dt; *J*= 8.6 and 2.0 Hz; 2H; H-5; H-9); 6.38 (d; *J*= 15.6 Hz; 1H; H-2); 5,74 (s; NH); 3.39 (t; *J*= 7.2 Hz; 1H; H-1'); 3.36 (t; *J*= 7.2 Hz; 1H; H-1'); 1.64-1.50 (m; 2H; H-2'); 1.40-1.29 (m; 4H; H-3', H-4'); 0.91 (t; *J*= 7.0 Hz; 3H; H-5'). ^{13}C NMR (50 MHz; $CDCl_3$): δ 165.45 (C-1); 139.51 (C-3); 133.81 (C-4); 131.98 (C-6, C-8); 129.11 (C-5, C-9); 123.66 (C-7); 121.45 (C-2); 39.79 (C-1'); 29.32 (C-2'); 29.06 (C-3'); 22.34 (C-4'); 13.95 (C-5'). EI-MS (*m/z*) (%): 295 (10; M^+); 211 (75); 209 (74); 102 (100). HRESI-MS: $[M+H]^+$ (*m/z*) calculated: 298.0645; found: 298.0647.

3c: **(2*E*)-3-(4-Bromophenyl)-*N,N*-dibutylprop-2-enamide**

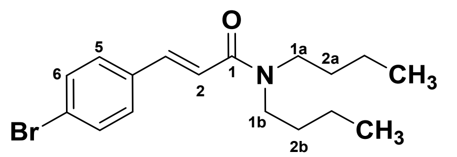

^{1}H NMR (300 MHz; CD_3OD): δ 7.54 (dt; *J*= 8.7 and 2.0; Hz; 2H; H-6; H-8) ; 7.48 (dt; *J*= 8.4 and 2.0 Hz; 2H; H-5, H-9); 7.43 (d; *J*=16.3 Hz; 1H; H-3); 6.51 (d; *J*= 16.3 Hz; 1H; H-2); 3.00-2.95 (m; 4H; H-1a; H-1b); 1.66 (m; 4H; H-2a; H-2b); 1.44 (m; 4H; H-3a; H-3b); 0.98 (m; 6H; H-4a; H-4b).^{13}C NMR (75 MHz; CD_3OD): δ 172.73 (C1); 139.02 (C3); 134.76 (C4); 131.60 (C6, C8); 128.91 (C5, C9); 124.78 (C7); 122.65 (C2); 48.48 (C1a); 47.33 (C1b); 27.88 (C2a, C2b); 19.46 (C3a, C3b); 12.54 (C4a, C4b). EI-MS (*m/z*) (%): 337 (5; M^+); 211 (91); 209 (100); 102 (93); 44 (83). HRESI-MS: $[M+H]^+$ (*m/z*) calculated: 338.1114; found: 338.1116.

4a: (2*E*)-3-(2*H*-1,3-Benzodioxol-5-yl)-1-(morpholin-4-yl)prop-2-en-1-one

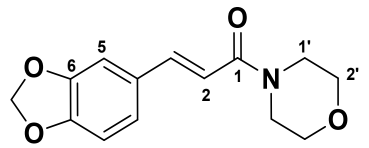

^{1}H NMR (300 MHz; $CDCl_3$): δ 7.62 (d; *J*= 15.3 Hz; 1H, H-3); 7.03 (d; *J*= 1.8 Hz; 1H; H-5); 7.02-6.98 (dd; *J*= 8.2 and 1.8 Hz; 1H; H-9); 6.81 (d; *J*= 8.2 Hz; 1H; H-8); 6.67 (d; *J*= 15.3 Hz; 1H; H-2); 5.99 (s; 2H; OCH_2O); 3.72 (s; 8H; H1'; H2'; H4'; H5'). ^{13}C NMR (75 MHz; $CDCl_3$): δ 165.63 (C-1); 149.06 (C-6); 148.20 (C-7); 142.94 (C-3); 129.48 (C-4); 123.84 (C9); 114.37 (C-2); 108.48 (C-8); 106.26 (C-5); 101.41 (OCH_2O); 66.80 (C-2', C-4'); 46.19 (C-1'); 42.49 (C-5'). EI-MS (*m/z*) (%): 261 (57; M^+); 176 (32); 175 (100); 145 (81); 117 (37); 89 (47). HRESI-MS: $[M+H]^+$ (*m/z*) calculated: 262.1074; found: 262.1072.

4b: (2*E*)-3-(2*H*-1,3-Benzodioxol-5-yl)-*N,N*-dibutylprop-2-enamide

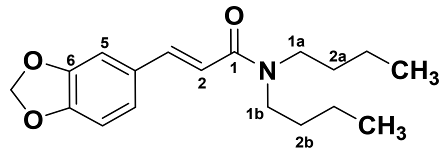

^{1}H NMR (200 MHz; $CDCl_3$): δ 7.42 (d; *J*= 15.8 Hz; 1H; H-3); 7.03 (d; *J*= 1.6 Hz; 1H; H-5); 6.99-6.94 (dd; *J*= 8.0 and 1.8 Hz; 1H; H-9); 6.79 (d; *J*= 8.0 Hz; 1H; H-8); 6.32 (d; *J*=15.8 Hz; 1H; H-2); 5.99 (s. 2H; OCH_2O); 2.90 (m; 4H; H-1a; H-1b); 1.93-1.77 (m; 4H; H-2a, H-2b); 1.50-1.33 (m; 4H; H-3a; H-3b); 0.95 (m; 6H; H-4a; H-4b).^{13}C NMR (50 MHz; $CDCl_3$): δ 172.74 (C-1); 148.43 (C-6); 147.96 (C-7); 140.51 (C-3); 129.90 (C-4); 123.13 (C-9); 122.16 (C-2); 108.22 (C-8); 106.25 (C-5); 101.11 (OCH_2O); 47.37 (C-1a; C-1b); 27.76 (C-2a; C-2b); 19.98 (C-3a; C-3b); 13.38 (C-4a, C-4b). EI-MS (*m/z*) (%): 303 (18; M^+); 175 (100); 145 (42); 89 (26). HRESI-MS: $[M+H]^+$ (*m/z*) calculated: 304.1907; found: 304.1902.

5a: **(2*E*)-*N,N*-Dibutyl-3-phenylprop-2-enamide**

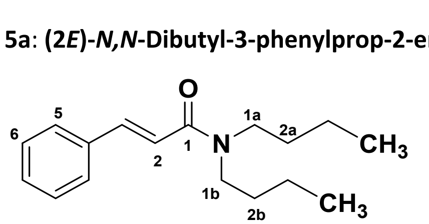

^{1}H NMR (200 MHz; $CDCl_3$): δ 7.70 (d; *J*= 15.4 Hz; 1H; H-3) 7.53-7.49 (m; 2H; H-5; H-9); 7.41-7.32 (m; 3H; H-6; H7; H8); 6.84 (d; *J*= 15.4 Hz; 1H; H-2); 3.47-3.35 (m; 4H; H-1a; H-1b); 1.70-1.51 (m; 4H; H2-a; H2-b); 1.46-1.26 (m; 4H; H-3a; H-3b); 1.01-0.91 (m; 6H; H-4a; H-4b). ^{13}C NMR (50 MHz; $CDCl_3$): δ 165.81 (C-1); 141.96 (C-3); 135.36 (C-4); 129.22 (C-7); 128.58 (C-6, C-8); 127.52 (C-5, C-9); 117.67 (C-2); 47.76 (C-1a); 46.50 (C-1b); 31.79 (C-2a); 29.92 (C-2b); 20.14 (C-3a); 19.92 (C-3b); 13.74 (C-4a); 13.68 (C-4b). EI-MS (*m/z*) (%): 259 (7; M^+); 131 (100); 103 (33). HRESI-MS: $[M+H]^+$ (*m/z*) calculated: 260.2008; found: 260.2010.

5b: **(2*E*)-*N*-Pentyl-3-phenylprop-2-enamide**

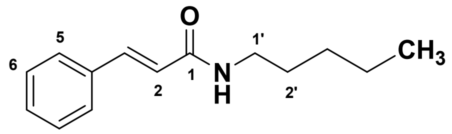

^{1}H NMR (300 MHz; $CDCl_3$): δ 7.62 (d; *J*= 15.6 Hz; 1H; H-3); 7.50-7.46 (m; 2H; H-5; H-9); 7.36-7.31 (m; 3H; H-6; H-7; H-8); 6.41 (d; *J*= 15.6 Hz; 1H; H-2); 5.82 (s; NH); 3.41-3.34 (m; 2H; H-1'); 1.62-1.52 (m; 2H; H-2'); 1.36-1.31 (m; 4H; H-3'; H-4'); 0.90 (t; *J*= 7.5 Hz; 3H; H-5'). ^{13}C NMR (75 MHz; $CDCl_3$): δ 166.09 (C-1); 140.96 (C-3); 135.15 (C-4); 129.76 (C-7); 128.99 (C-6; C-8); 127.95 (C-5; C-9); 121.12 (C2); 40.01 (C-1'); 29.60 (C-2'); 29.34 (C-3'); 22.60 (C-4'); 14.20 (C-5'). EI-MS (*m/z*) (%): 217 (7; M^+); 131 (100); 103 (45); 77 (25). HRESI-MS: $[M+H]^+$ (*m/z*) calculated: 218.1539; found: 218.1538.

5c: **(2*E*)-1-(Morpholin-4-yl)-3-phenylprop-2-en-1-one**

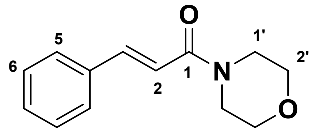

^{1}H NMR (300 MHz; $CDCl_3$): δ 7.70 (d; *J*=15.3 Hz; 1H; H-3); 7.54-7.50 (m; 2H; H-5; H-9); 7.40-7.34 (m; 3H; H-6; H-7; H-8); 3.72 (s; 8H; H-1', H-2'; H-4'; H-5'). ^{13}C NMR (75 MHz; $CDCl_3$): δ 165.48 (C-1); 143.10 (C-3); 135.05 (C-4); 129.66 (C-7); 128.74 (C-6, C-8); 127.70 (C-5, C-9); 116.49 (C-2); 66.78 (C-2'; C-4'); 46.17 (C-5'); 42.41 (C-1'). EI-MS (*m/z*) (%): 217 (18; M^+); 131 (100); 103 (60); 86 (26); 77 (28). HRESI-MS: $[M+H]^+$ (*m/z*) calculated: 218.1175; found: 218.1180.

5d: **1-[(2*E*)-3-Phenylprop-2-enoyl]pyrrolidin-2-one**

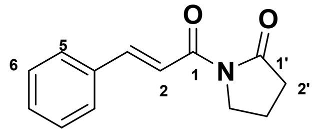

^{1}H NMR (300 MHz; $CDCl_3$): δ 7.95 (d; *J*= 15.9 Hz; 1H; H-2); 7.83 (d; *J*=15.9 Hz; 1H; H-3); 7.63-7.59 (m; 2H; H-5; H-9); 7.40-7.36 (m; 3H; H-6; H-7; H-8); 3.92 (t; *J*= 7.5 Hz; 2H; H-4'); 2.65 (t; *J*= 9.0 Hz; 2H; H-2'); 2.07 (m; 2H; H-3'). ^{13}C NMR (75 MHz; $CDCl_3$): δ 175.64 (C-1'); 166.30 (C-1); 145.47 (C-3); 134.87 (C-4); 130.32 (C-7); 128.78 (C-6; C-8); 128.48 (C-5; C-9); 119.00 (C-2); 45.84 (C-4'); 33.96 (C-2'); 17.19 (C-3'). EI-MS (*m/z*) (%): 215 (26; M^+); 131 (100); 103 (57); 77 (32). HRESI-MS: $[M+H]^+$ (*m/z*) calculated: 216.1019; found: 216.1015.

6a: **(2*E*,4*E*)-5-(2*H*-1,3-Benzodioxol-5-yl)-1-(piperidin-1-yl)penta-2,4-dien-1-one**

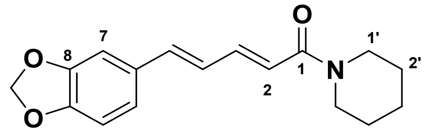

^{1}H NMR (300 MHz; $CDCl_3$): δ 7.44-7.36 (m; 1H; H-3); 6.97 (d; *J*= 1.8 Hz; 1H; H-7); 6.90-6.87 (dd; *J*= 8.1 and 1.8 Hz; 1H; H11); 6.78 (d; *J*= 8.1 Hz; 1H; H-10); 6.75- 6.72 (m; 2H; H-4; H-5); 6.43 (d; *J*=14.4 Hz; 1H; H-2); 5.97 (s; 2H; OCH_2O); 3.62-3.47 (m; 4H; H-1'; H-5'); 1.67-1.54 (m; 6H; H-2'; H-3'; H-4'). ^{13}C NMR (75 MHz; $CDCl_3$): δ 165.34 (C-1); 148.03 (C-9); 148.11 (C-8); 142.38 (C-3); 138.12 (C-5); 130.94 (C-6); 125.29 (C-4); 122.40 (C-11); 120.00 (C-2); 108.40 (C-10); 105.59 (C-7); 101.19 (OCH_2O); 46.83 (C-1'); 43.17 (C-5'); 26.63 (C-2'); 25.59 (C-4'); 24.59 (C-3'). EI-MS (*m/z*) (%): 285 (63; M^+); 202 (25); 201 (87); 174 (25); 173 (42); 172 (25); 143 (35); 116 (25); 115 (100); 84 (35). HRESI-MS: $[M+H]^+$ (*m/z*) calculated: 286.1438; found: 286.1441.

6b: **(2*E*,4*E*)-5-(2*H*-1,3-Benzodioxol-5-yl)-*N*-pentylpenta-2,4-dienamide**

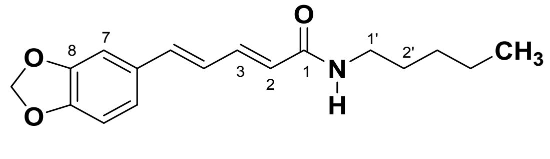

^{1}H NMR (300 MHz; $CDCl_3$): δ 7.39-7.31 (dd; *J*= 15.0 and 14.8 Hz; 1H; H-3); 6.97 (d; *J*= 1.8 Hz; 1H; H-7); 6.90-6.86 (dd; *J*= 8.1 and 1.8 Hz; 1H, H-11); 6.79-6.74 (m; 2H; H-4; H-5); 6.70-6.62 (m; 1H; H-10); 5.97 (s; 2H; OCH_2O); 5.91 (d; *J*= 14.8 Hz; 1H; H-2); 5.63 (s; NH); 3.38-3.31 (m; 2H; H-1'); 1.59-1.50 (m; 2H; H-2'); 1.37-1.30 (m; 4H; H-3', H-4'); 0.90 (t; *J*= 7.4 Hz; 3H; H-5'). ^{13}C NMR (75 MHz; $CDCl_3$): δ 166.07 (C-1); 148.11 (C-8. C-9); 140.79 (C-3); 138.68 (C-5); 130.81 (C-6); 124.62 (C-4); 123.22 (C-11); 122.49 (C-2); 108.40 (C-10); 105.63 (C-7); 101.22 (OCH_2O); 39.64 (C-1'); 29.32 (C-2'); 29.05 (C-3'); 22.32 (C-4'); 13.92 (C-5'). EI-MS (*m/z*) (%): 287 (55; M^+); 201 (57); 174 (40); 173 (100); 172 (30); 143 (40); 116 (25); 115 (99); 101 (24); 96 (25). HRESI-MS: $[M+H]^+$ (*m/z*) calculated: 288.1594; found: 288.1596.

7a: (2*E*,4*E*)-5-(4-Methoxyphenyl)-*N*-pentylpenta-2,4-dienamide

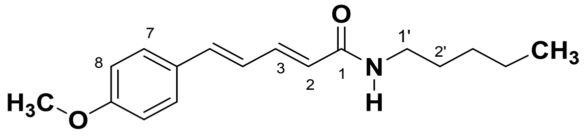

^{1}H NMR (200 MHz; $CDCl_3$): δ 7.38 (d; *J*= 8.8 Hz; 2H; H-7, H-11); 7.24-7.50 (m; 1H; H-3); 6.83 (d; *J* = 8.8 Hz; 2H; H-8, H-10); 6.89-6.63 (m. 2H; H-4, H-5); 5.94 (d; *J*= 14.9 Hz; 1H; H-2); 5.91 (s; NH); 3.80 (s; 3H; OMe-9); 3.34 (m; 2H; H-1'); 1.54 (m; 2H; H-2'); 1.36-1.25 (m; 4H; H-3', H-4'); 0.90 (t; *J* = 7.0 Hz; 3H; H-5'). ^{13}C NMR (50 MHz; $CDCl_3$): δ 166.3 (C-1); 160.00 (C-9); 141.00 (C-3); 138.70 (C-5); 129.10 (C-6); 128.30 (C-7, C-11); 124.30 (C-4); 122.90 (C-2); 114.10 (C-8, C-10); 55.20 (OMe-9); 39.70 (C-1'); 29.30 (C-2'); 29.10 (C-3'); 22.30 (C-4'); 13.90 (C-5'). EI-MS (*m/z*) (%): 273 (87; M^+); 204 (26); 188 (35); 187 (100); 166 (25); 160 (31); 159 (62); 148 (40); 144 (60); 128 (32); 127 (26); 121 (42); 116 (32); 115 (60); 96 (37). HRESI-MS: $[M+H]^+$ (*m/z*) calculated: 274.1802; found: 274.1800.

7b: (2*E*,4*E*)-*N,N*-Dibutyl-5-(4-methoxyphenyl)penta-2,4-dienamide

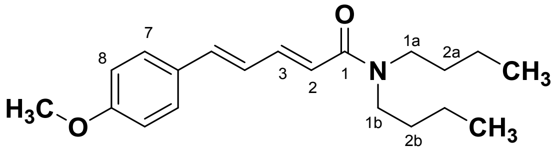

^{1}H NMR (200 MHz; $CDCl_3$): δ 7.43-7.51 (m; 1H; H-3); 7.39 (d; *J*= 8.8 Hz; 2H; H-7, H-11); 6.89 (d; *J*= 8.8 Hz; 2H; H8, H10); 6.87-6.63 (m; 2H; H-4, H-5); 6.35 (d; *J*= 14.5 Hz; 1H; H-2); 3.80 (s; 3H; OMe-9); 3.40 (t; *J*= 7.4 Hz; 2H; H-1a); 3.32 (t; *J*= 7.4 Hz; 2H; H-1b); 1.64-1.48 (m; 4H; H-2a, H-2b); 1.36-1.25 (m; 4H; H-3a, H-3b); 0.96 (t; *J*= 7.0 Hz; 3H; H-4a); 0.93 (t; *J*= 7.0 Hz; 3H; H-4b). ^{13}C NMR (50 MHz; $CDCl_3$): δ 166.11 (C-1); 159.91 (C-9); 142.61 (C-5); 138.20 (C-3); 129.09 (C-6); 128.20 (C-7, C-11); 124.89 (C-4); 119.67 (C-2); 114.00 (C-8, C-10); 55.10 (OMe-9); 47.78 (C-1a); 46.44 (C-1b); 31.81 (C-2a); 30.00 (C-2b); 20.10 (C-3a); 20.00 (C-3b); 13.80 (C-4a); 13.70 (C-4b). EI-MS (*m/z*) (%): 315 (26; M^+); 187 (100); 144 (27); 128 (27); 115 (27); 44 (35). HRESI-MS: $[M+H]^+$ (*m/z*) calculated: 316.2271; found: 316.2258.

7c: **(2*E*,4*E*)-5-(4-Methoxyphenyl)-1-(piperidin-1-yl)penta-2,4-dien-1-one**

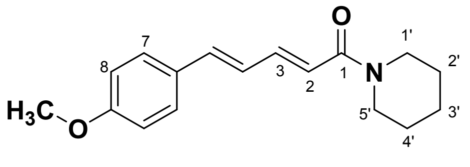

^{1}H NMR (200 MHz; $CDCl_3$): δ 7.51-7.29 (m; 1H; H-3); 7.38 (d; *J*= 8.4 Hz; 2H; H-7, H-11); 6.87 (d; *J*= 8.4 Hz; 2H; H-8, H-10); 6.80-6.70 (m; 2H; H-4, H-5); 6.43 (d; *J*= 14.5 Hz; 1H; H-2); 3.80 (s; 3H; OMe-9); 3.61 (m; 2H; H-5'); 3.52 (m; 2H; H-1'); 1.61 (m; 6H; H-2', H-3', H-4'). ^{13}C NMR (50 MHz; $CDCl_3$): δ 165.30 (C-1); 159.80 (C-9); 142.60 (C-5); 138.00 (C-3); 129.10 (C-6); 128.20 (C-7, C-11); 124.80 (C-4); 119.50 (C-2); 114.00 (C-8, C-10); 55.10 (OMe-9); 46.70 (C-5'); 43.10 (C-1'); 26.50 (C-4'); 25.40 (C-2'); 24.50 (C-3'). EI-MS (*m/z*) (%): 271 (67; M^+); 188 (37); 187 (100); 186 (25); 159 (30); 144 (40); 137 (25); 115 (42); 84 (35). HRESI-MS: $[M+H]^+$ (*m/z*) calculated: 272.1645; found: 272.1646.

8a: **(2*E*,4*E*)-5-(4-Bromophenyl)-*N*-pentylpenta-2,4-dienamide**

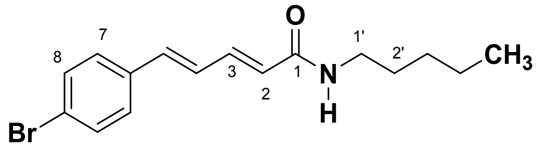

^{1}H NMR (300 MHz; $CDCl_3$): δ 7.44 (dt; *J*= 8.4 and 2.0 Hz; 2H; H-8, H-10); 7.40-7.32 (m; 1H; H-5) 7.27 (dt; *J*= 8.4 and 2.0 Hz; 2H; H-7; H-11); 6.84 (d; *J* = 15.6; 1H; H-3); 6.75 (d; *J*= 15.6 Hz; 1H; H-4); 6.00 (d; *J* = 15.6 Hz; 1H; H-2); 5.81 (s, NH); 3.38-3.31 (m; 2H; H-1'); 1.55 (m; 2H; H-2'); 1.35-1.30 (m; 4H; H-3'; H-4'); 0.90 (t; *J*= 7.3 Hz; 3H; H-5'). ^{13}C NMR (75 MHz; $CDCl_3$): δ 165.84 (C-1); 140.28 (C-5); 137.51 (C-3); 135.19 (C-6); 131.83 (C-8, C-10); 128.30 (C-7, C-11); 126.99 (C-4); 124.67 (C-9); 122.49 (C-2); 39.70 (C-1'); 29.30 (C-2'); 29.06 (C-3'); 22.33 (C-4'); 13.94 (C-5'). EI-MS (*m/z*) (%): 321 (18; M^+); 237 (35); 235 (37); 156 (25); 129 (27); 128 (100); 127 (26); 96 (45). HRESI-MS: $[M+H]^+$ (*m/z*) calculated: 322.0801; found: 322.0801.

8b: (2*E*,4*E*)-5-(4-Bromophenyl)-1-(piperidin-1-yl)penta-2,4-dien-1-one

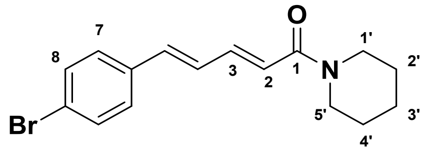

^{1}H NMR (300 MHz; $CDCl_3$): δ 7.76 (dt; *J*= 8.4 and 1.8 Hz; 2H; H-8; H-10); 7.44-7.36 (m; 1H; H-3); 7.30 (dt; *J*= 8.4 and 1.8 Hz; 2H; H-7; H-11); 6.93-6.74 (m; 2H; H-4, H-5); 6.50 (d; *J*= 15.0 Hz; 1H; H-2); 3.63 (m; 2H; H-1'); 3.54 (m; 2H; H-5'); 1.67-1.56 (m; 6H; H-2'; H-3'; H-4'); ^{13}C NMR (75 MHz; $CDCl_3$): δ 165.11 (C-1); 141.82 (C-5); 136.92 (C-3); 135.29 (C-6); 131.76 (C-8, C-10); 128.23 (C-7; C-11); 127.58 (C-4); 122.29 (C-9); 121.43 (C-2); 46.87 (C-1'); 43.21 (C-5'); 26.65 (C-2'); 25.52 (C-4'); 24.52 (C-3'). EI-MS (*m/z*) (%): 319 (25; M^+); 237 (25); 235 (25); 156 (27); 138 (27); 129 (28); 128 (100); 84 (74). HRESI-MS: $[M+H]^+$ (*m/z*) calculated: 320.0645; found: 320.0649.

9: 1-[3-(3,4,5-Trimethoxyphenyl)propanoyl]piperidin-2-one

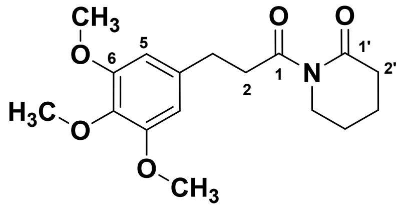

^{1}H NMR (200 MHz; $CDCl_3$): δ 6.46 (s; 2H; H5; H-9); 3.90 (s; 6H; OMe-6 and 8); 3.80 (s; 3H; OMe-7); 3.72 (m; 2H; H-5'); 3.32 (t; *J*= 7.0 Hz; 2H. H-3); 2.92 (t; *J*= 7.0 Hz; 2H; H-2); 2.83 (m; 2H; H-2'); 1.80 (m; 4H; H-3'.H-4'). ^{13}C NMR (50 MHz; $CDCl_3$): δ 175.95 (C-1); 173.25 (C-1'); 152.92 (C-6,C-8); 136.85 (C-4); 136.00 (C-7); 105.32 (C-5. C-9); 60.66 (OMe-7); 55.91 (OMe-6 and 8); 43.89 (C-2); 41.16 (C-2'); 34.73 (C-5'); 31.39 (C-3); 22.27 (C-4'); 20.11 (C-3'). EI-MS (*m/z*) (%): 321 (35; M^+); 222 (100); 194 (40); 191 (25); 181 (25); 179 (50). HRESI-MS: $[M+H]^+$ (*m/z*) calculated: 322.1649; found: 322.1649.

10: (2*E*,4*E*)-5-(2*H*-1,3-Benzodioxol-5-yl)-1-(piperidin-1-yl)penta-2,4-dien-1-one

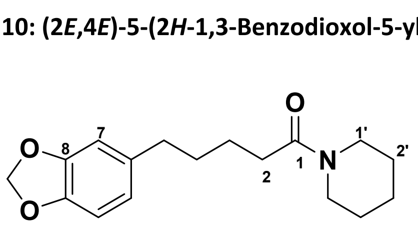

^{1}H NMR (300MHz; $CDCl_3$): δ 6.70 (d; *J*= 7.8 Hz; 1H; H-10); 6.66 (d; *J*= 1.5 Hz; 1H; H-7); 6.63- 6.59 (dd; *J*= 7.8 and 1.5 Hz; 1H; H11); 5.89 (s; 2H; OCH_2O); 3.53 (t; *J*= 7.1 Hz; 2H; H-1'); 3.35 (t; *J*= 7.2 Hz; 2H; H-5'); 2.55 (t; *J*= 7.5 Hz; 2H; H-5); 2.32 (t; *J*= 7.5 Hz; 2H; H-2); 1.64-1.61 (m; 6H; H-3; H-4; H-3'); 1.54-1.50 (m; 4H; H-2'; H-4'). ^{13}C NMR (75 MHz; $CDCl_3$): δ 170.97 (C-1); 147.27 (C-8); 145.27 (C-9); 135.94 (C-6); 120.86 (C-11); 108.62 (C-7); 107.81 (C-10); 100.47 (OCH_2O); 46.48 (C-1'); 42.40 (C-5'); 35.23 (C-5); 33.03 (C-2); 31.24 (C-4); 26.33 (C-3); 25.38 (C-3'); 24.71

(C-2'); 24.35 (C-4'). EI-MS (m/z) (%): 289 (35; M^+); 204 (25); 140 (31); 127 (100); 112 (52); 86 (25); 84 (35). HRESI-MS: $[M+H]^+$ (m/z) calculated: 290.1751; found: 290.1752.

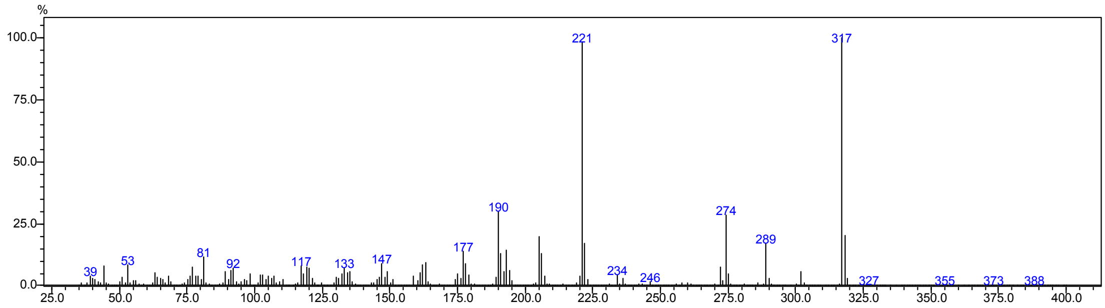

Fig S1 EI spectrum of **1a** (1-[(2*E*)-3-(3,4,5-trimethoxyphenyl)prop-2-enoyl]-1,2,5,6-tetrahydropyridin-2-one)

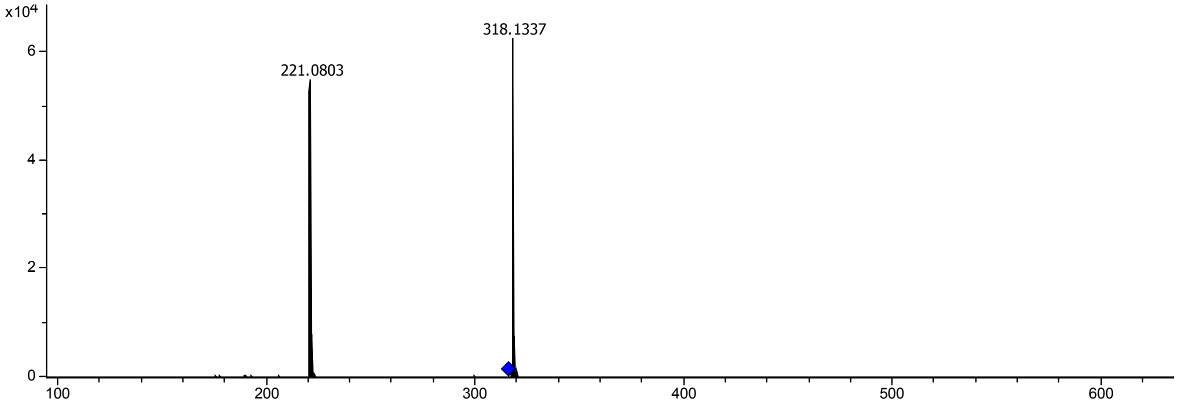

Fig S2 HRESI spectrum of **1a** (1-[(2*E*)-3-(3,4,5-trimethoxyphenyl)prop-2-enoyl]-1,2,5,6-tetrahydropyridin-2-one)

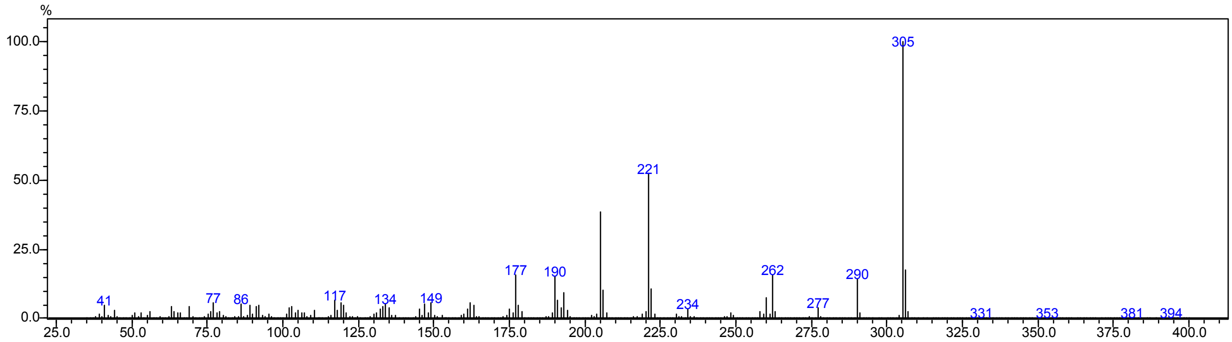

Fig S3 EI spectrum of **1b** (1-[(2*E*)-3-(3,4,5-trimethoxyphenyl)prop-2-enoyl]pyrrolidin-2-one

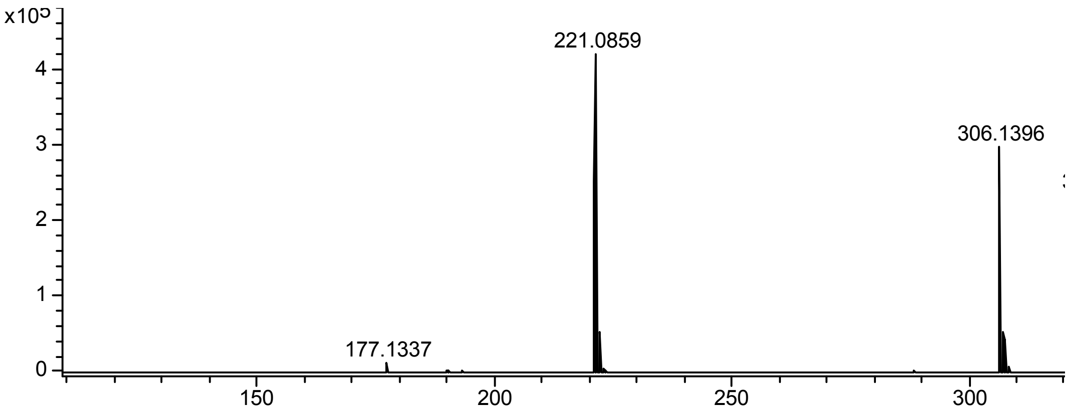

Fig S4 HRESI spectrum of **1b** (1-[(2*E*)-3-(3,4,5-trimethoxyphenyl)prop-2-enoyl]pyrrolidin-2-one

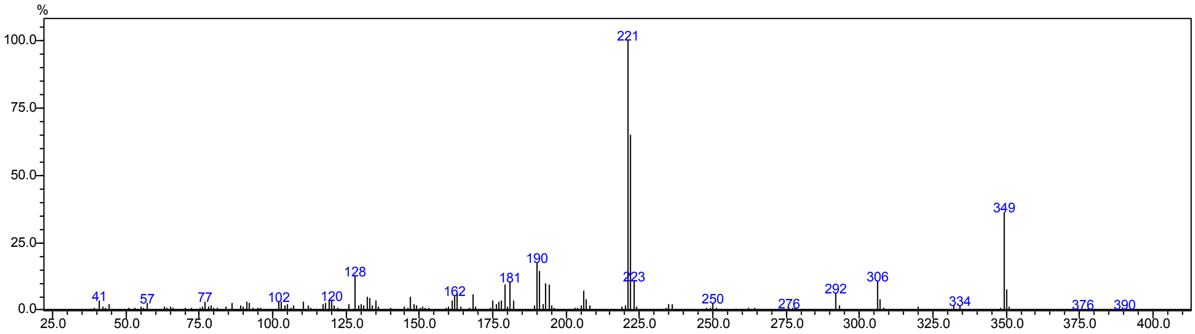

Fig S5 EI spectrum of **1c** (2E)-*N,N*-dibutyl-3-(3,4,5-trimethoxyphenyl)prop-2-enamide

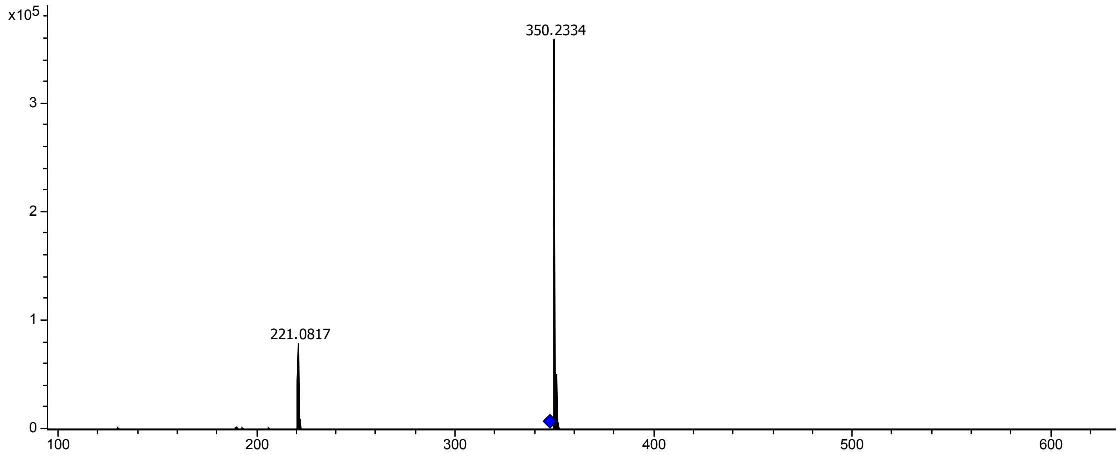

Fig S6 HRESI spectrum of **1c** (2E)-*N,N*-dibutyl-3-(3,4,5-trimethoxyphenyl)prop-2-enamide

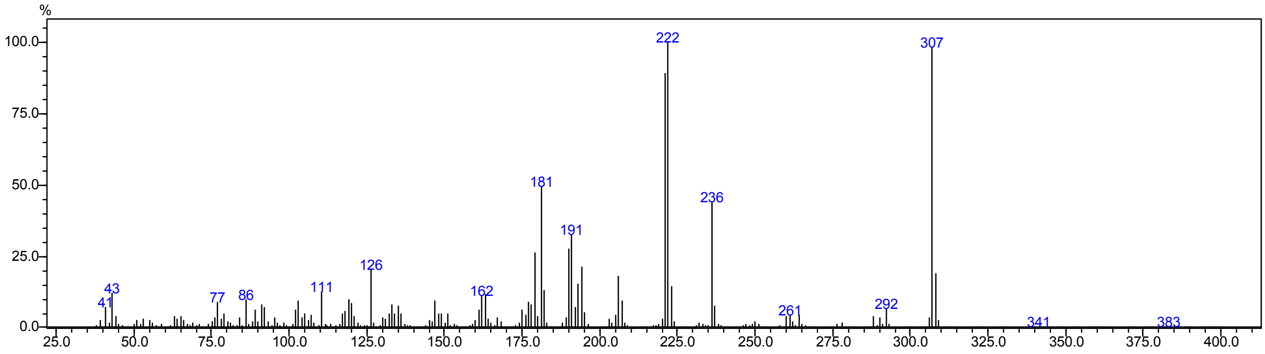

Fig S7 EI spectrum of **1d** (2*E*)-*N*-pentyl-3-(3,4,5-trimethoxyphenyl)prop-2-enamide

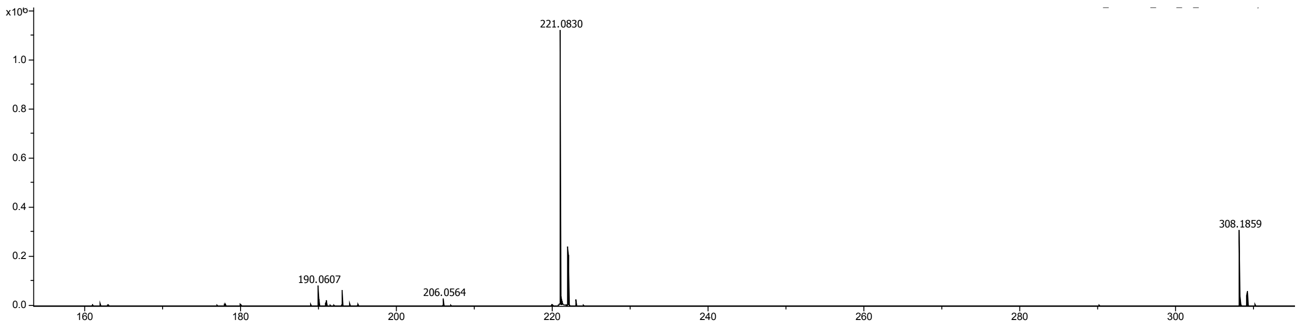

Fig S8 HRESI spectrum of **1d** (2*E*)-*N*-pentyl-3-(3,4,5-trimethoxyphenyl)prop-2-enamide

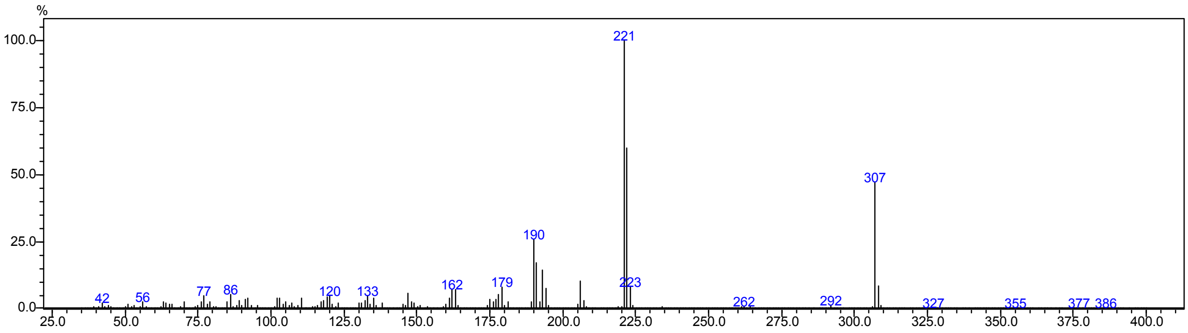

Fig S9 EI spectrum of **1e** (2*E*)-1-(morpholin-4-yl)-3-(3,4,5-trimethoxyphenyl)prop-2-en-1-one

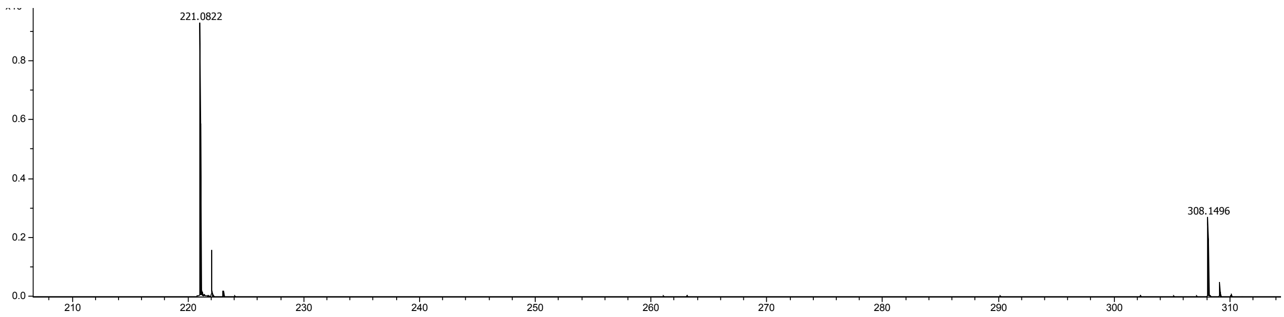

Fig S10 HRESI spectrum of **1e** (2*E*)-1-(morpholin-4-yl)-3-(3,4,5-trimethoxyphenyl)prop-2-en-1-one

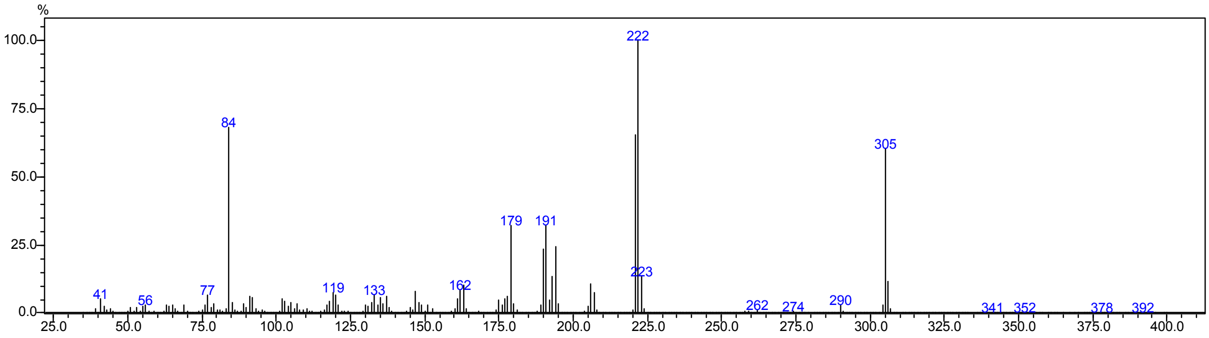

Fig S11 EI spectrum of **1f** (2*E*)-1-(piperidin-1-yl)-3-(3,4,5-trimethoxyphenyl)prop-2-en-1-one

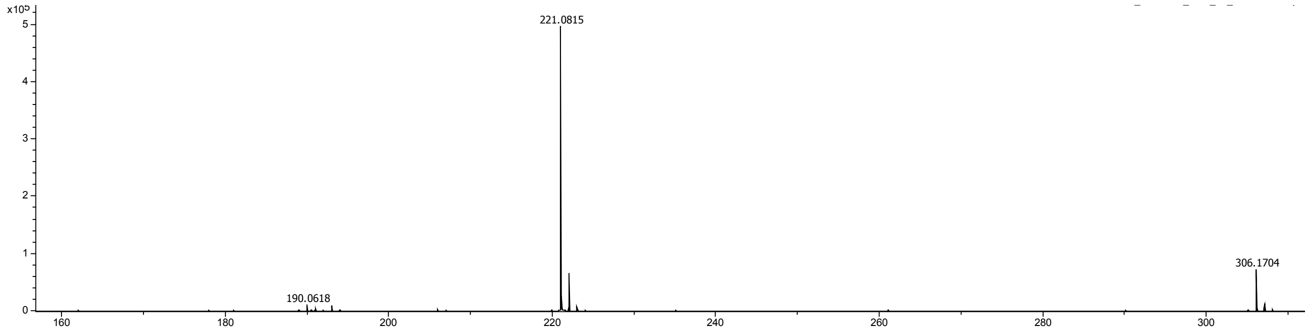

Fig S12 HRESI spectrum of **1f** (2*E*)-1-(piperidin-1-yl)-3-(3,4,5-trimethoxyphenyl)prop-2-en-1-one

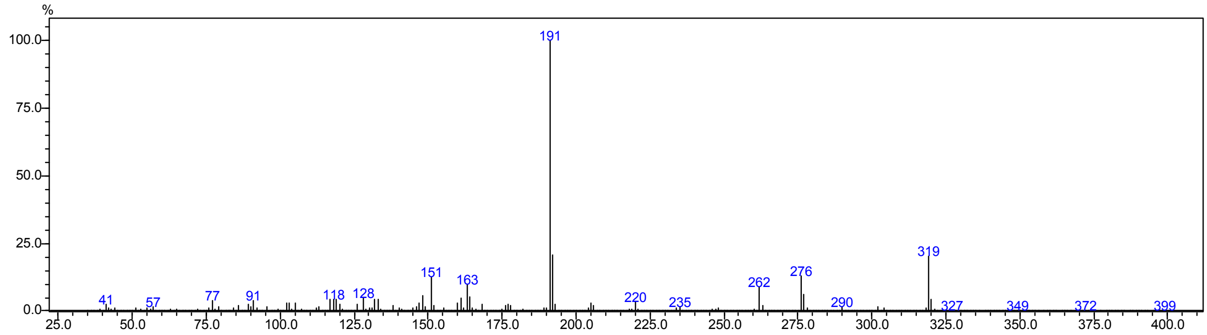

Fig S13 EI spectrum of **2a** (2*E*)-*N,N*-dibutyl-3-(3,4-dimethoxyphenyl)prop-2-enamide

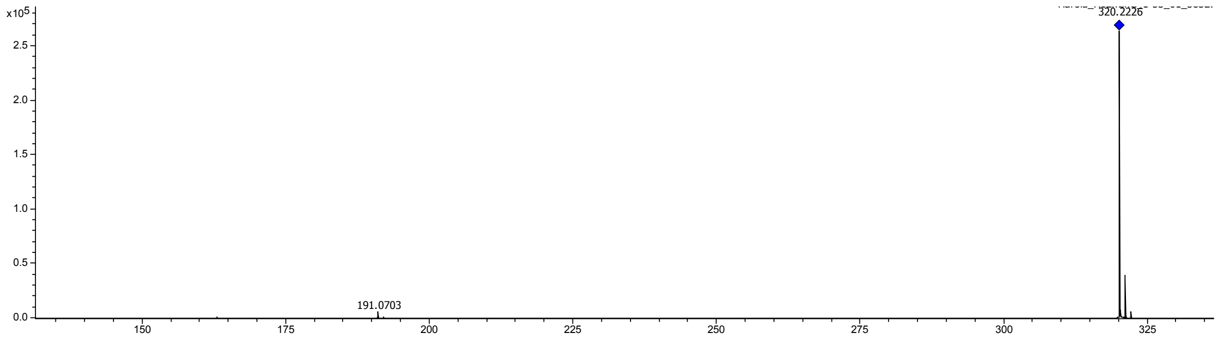

Fig S14 HRESI spectrum of **2a** (2*E*)-*N,N*-dibutyl-3-(3,4-dimethoxyphenyl)prop-2-enamide

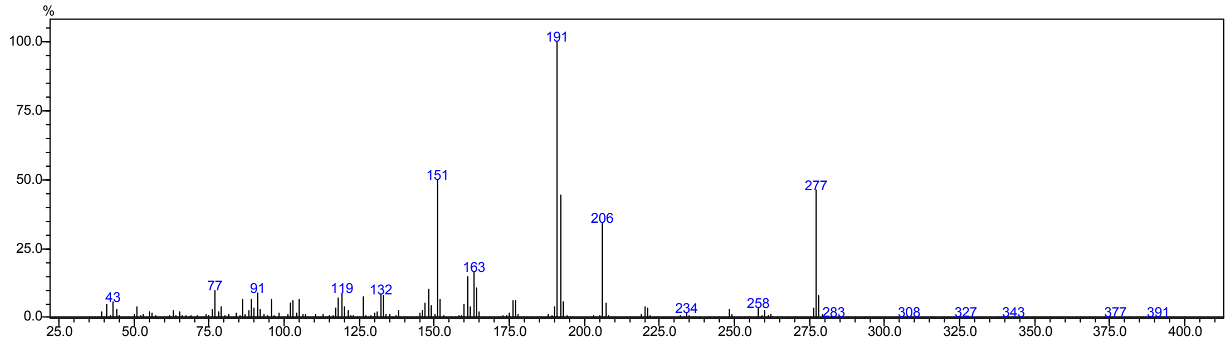

Fig S15 EI spectrum of **2b** (2*E*)-3-(3,4-dimethoxyphenyl)-*N*-pentylprop-2-enamide

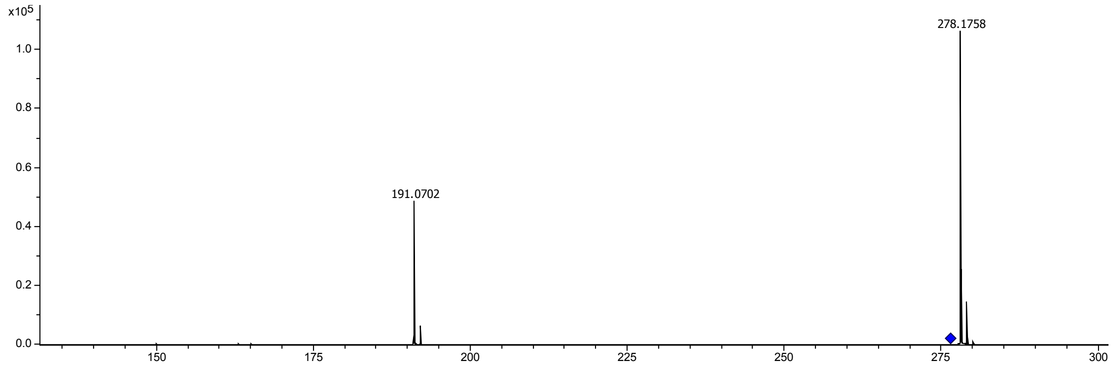

Fig S16 HRESI spectrum of **2b** (2*E*)-3-(3,4-dimethoxyphenyl)-*N*-pentylprop-2-enamide

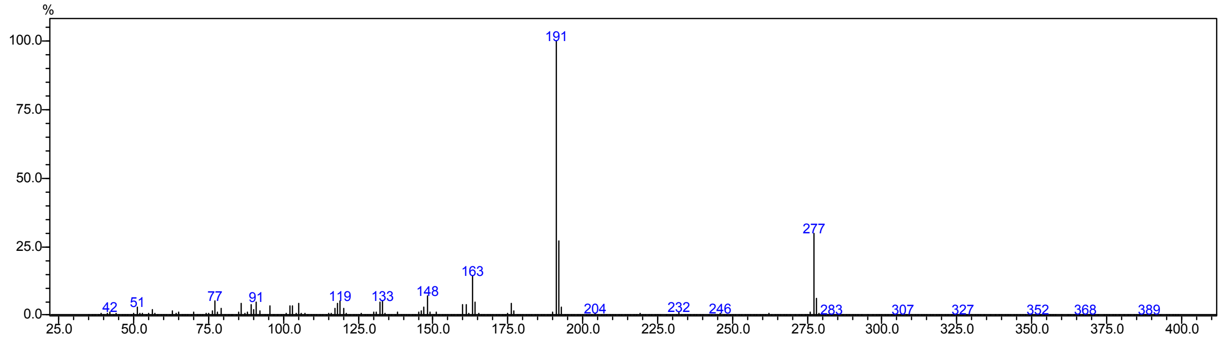

Fig S17 EI spectrum of **2c** (2*E*)-3-(3,4-dimethoxyphenyl)-1-(morpholin-4-yl)prop-2-en-1-one

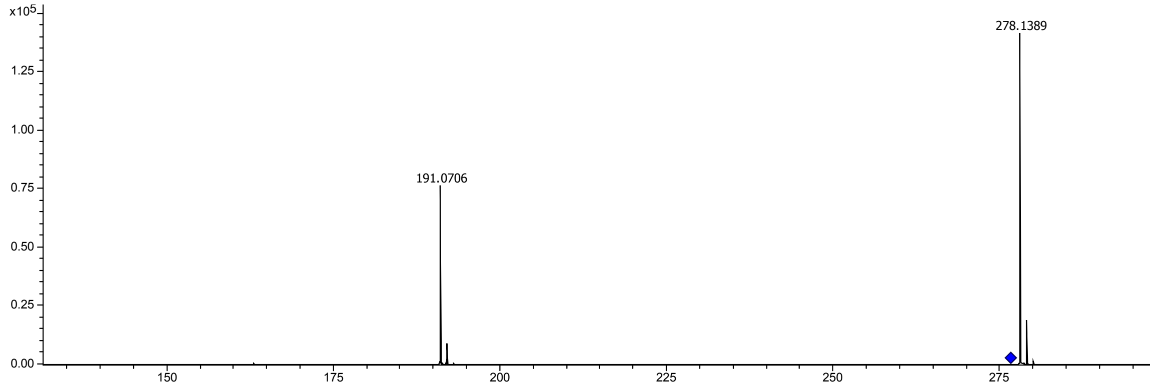

Fig S18 HRESI spectrum of **2c** (2*E*)-3-(3,4-dimethoxyphenyl)-1-(morpholin-4-yl)prop-2-en-1-one

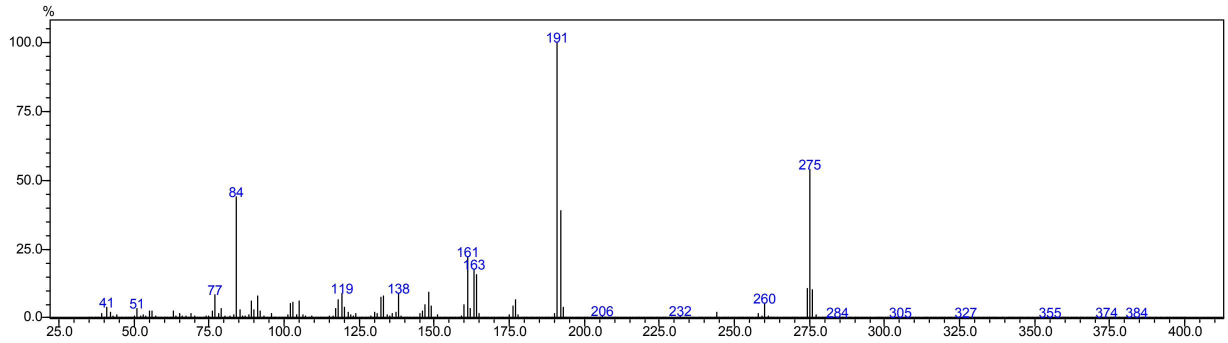

Fig S19 EI spectrum of **2d** (2*E*)-3-(3,4-dimethoxyphenyl)-1-(piperidin-1-yl)prop-2-en-1-one

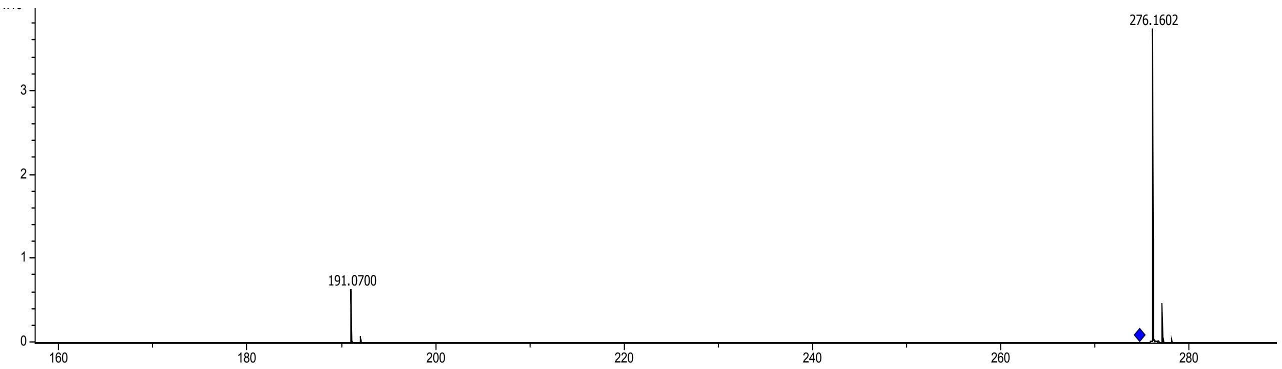

Fig S20 HRESI spectrum of **2d** (2*E*)-3-(3,4-dimethoxyphenyl)-1-(piperidin-1-yl)prop-2-en-1-one

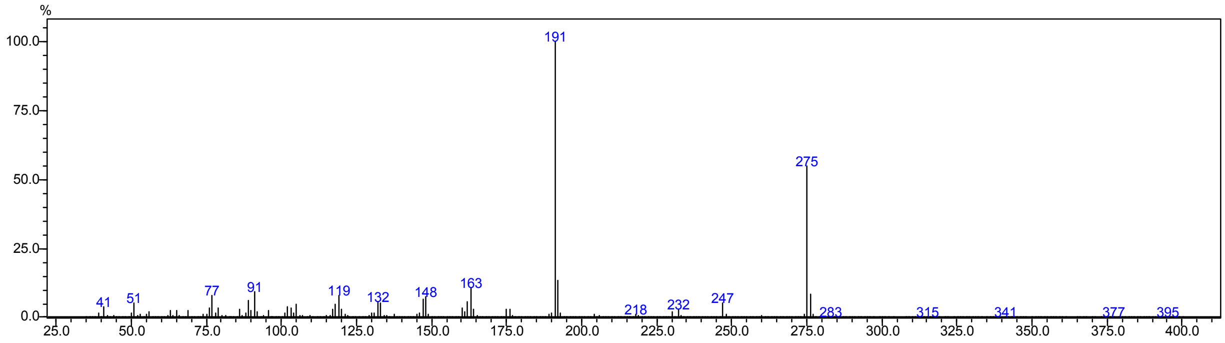

Fig S21 EI spectrum of **2e** 1-[(2*E*)-3-(3,4-dimethoxyphenyl)prop-2-enoyl]pyrrolidin-2-one

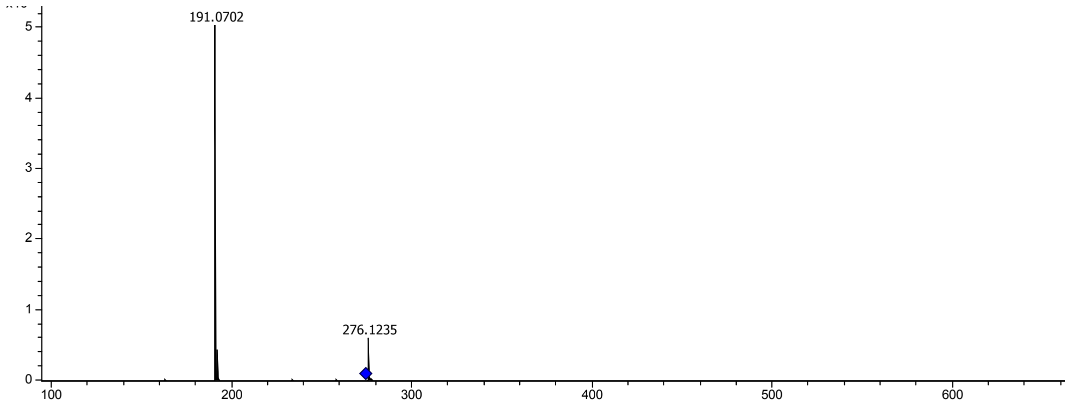

Fig S22 HRESI spectrum of **2e** 1-[(2*E*)-3-(3,4-dimethoxyphenyl)prop-2-enoyl]pyrrolidin-2-one

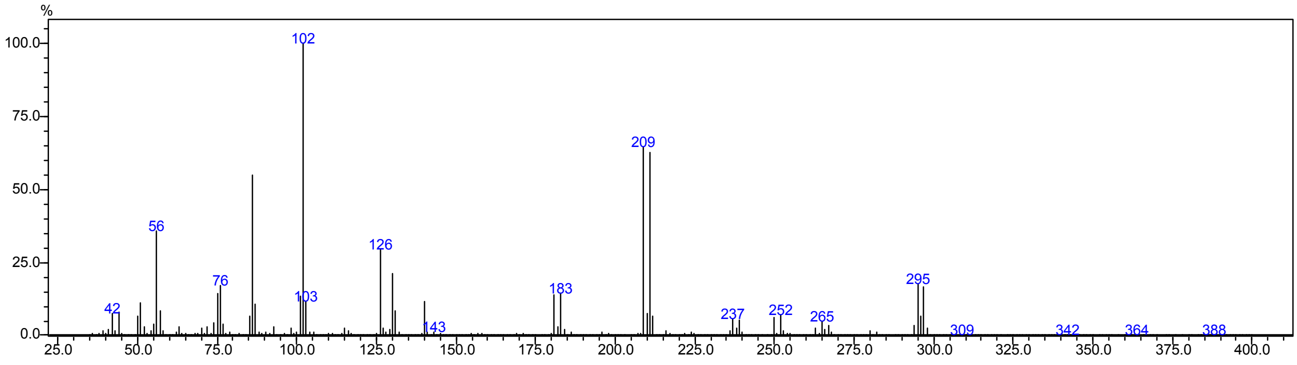

Fig S23 EI spectrum of **3a** (2*E*)-3-(4-bromophenyl)-1-(morpholin-4-yl)prop-2-en-1-one

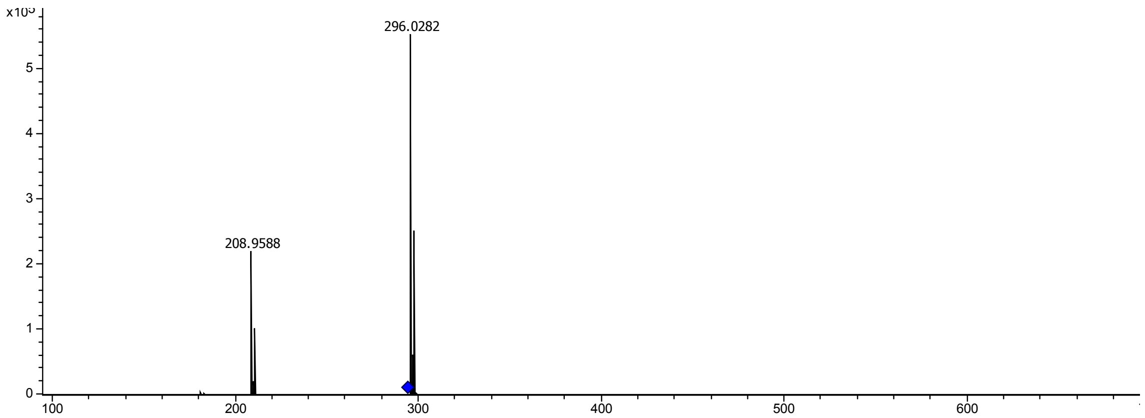

Fig S24 HRESI spectrum of **3a** (2*E*)-3-(4-bromophenyl)-1-(morpholin-4-yl)prop-2-en-1-one

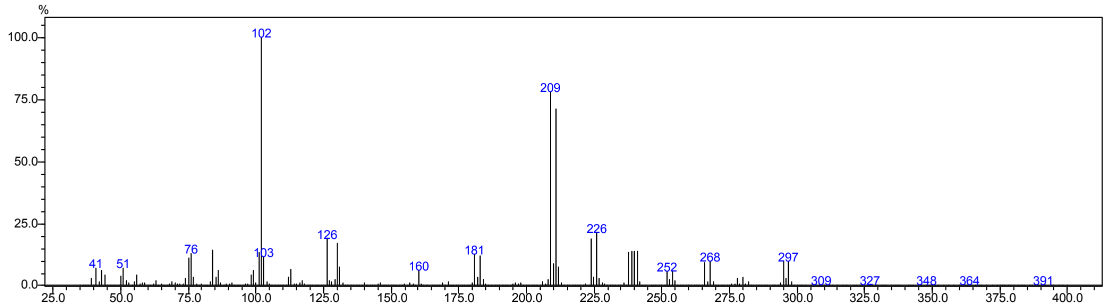

Fig S25 EI spectrum of **3b** (2*E*)-3-(4-bromophenyl)-*N*-pentylprop-2-enamide

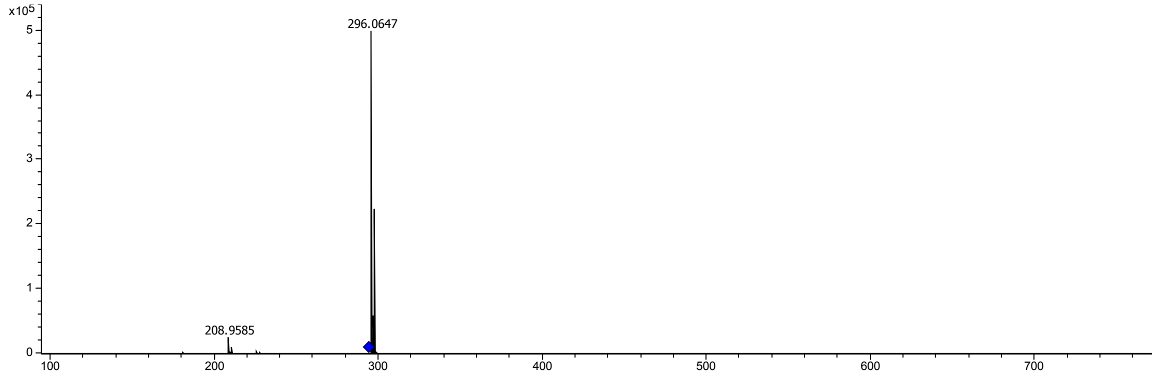

Fig S26 HRESI spectrum of **3b** (2*E*)-3-(4-bromophenyl)-*N*-pentylprop-2-enamide

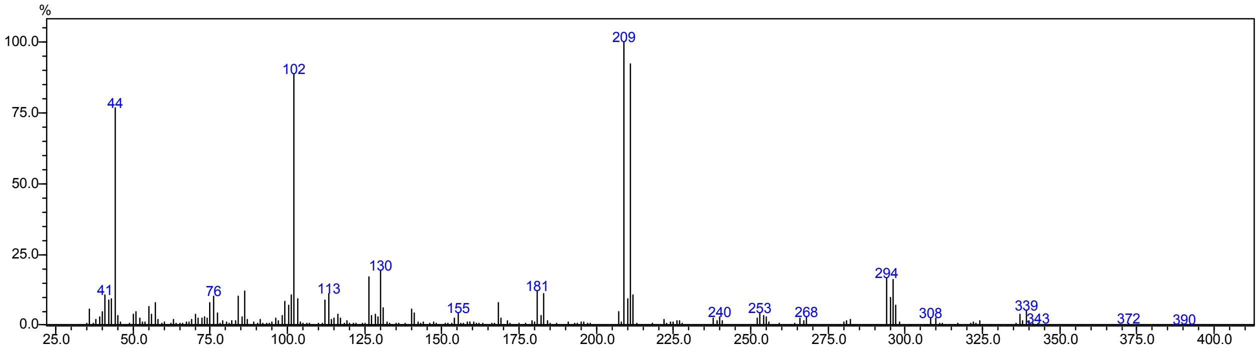

Fig S27 EI spectrum of **3c** (2*E*)-3-(4-bromophenyl)-*N,N*-dibutylprop-2-enamide

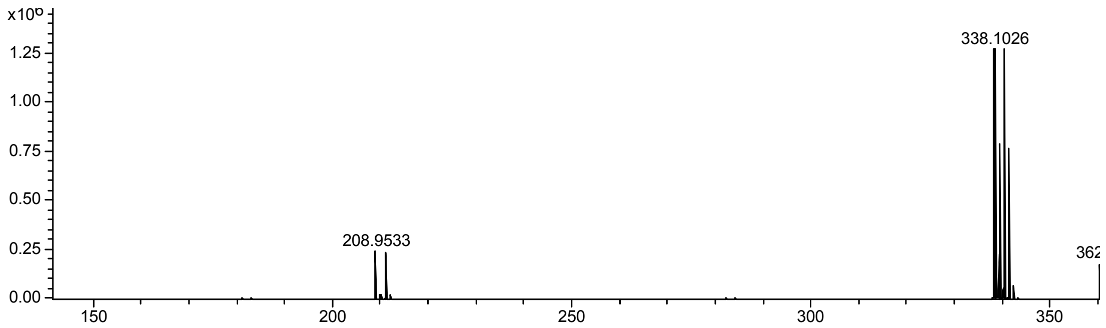

Fig S28 HRESI spectrum of **3c** (2*E*)-3-(4-bromophenyl)-*N,N*-dibutylprop-2-enamide

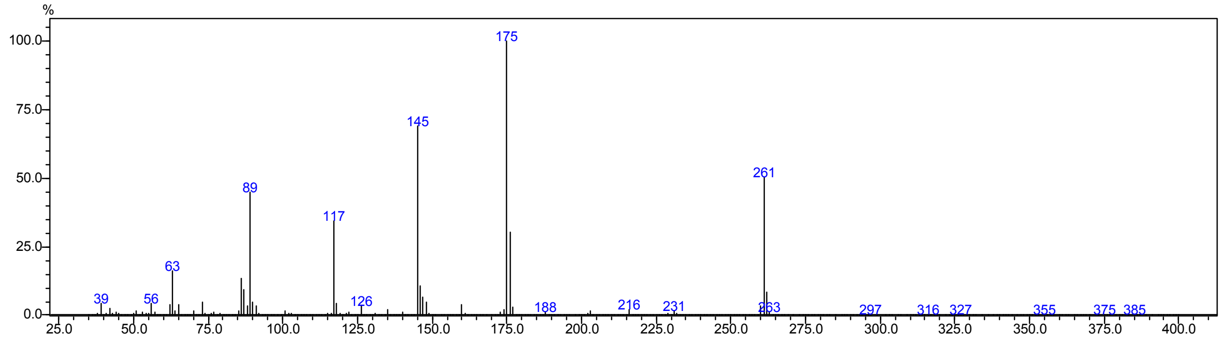

Fig S29 EI spectrum of **4a** (2*E*)-3-(2*H*-1,3-benzodioxol-5-yl)-1-(morpholin-4-yl)prop-2-en-1-one

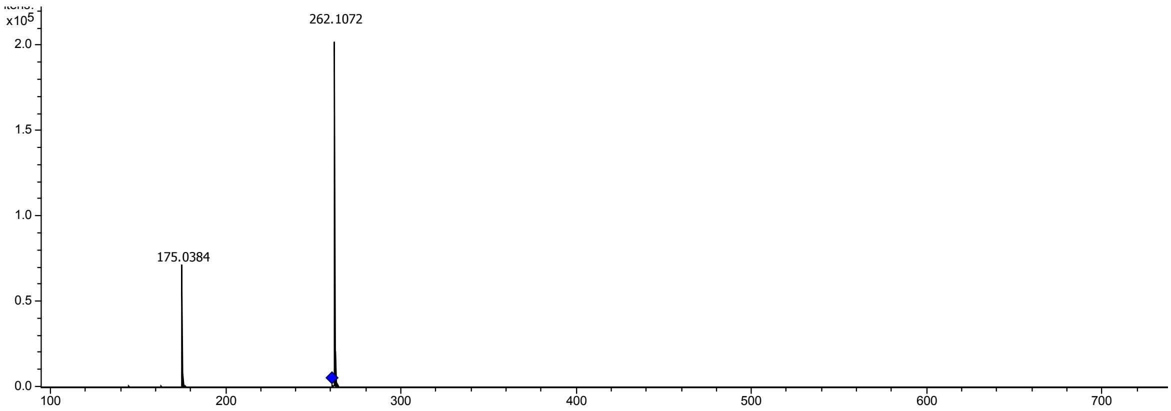

Fig S30 HRESI spectrum of **4a** (2*E*)-3-(2*H*-1,3-benzodioxol-5-yl)-1-(morpholin-4-yl)prop-2-en-1-one

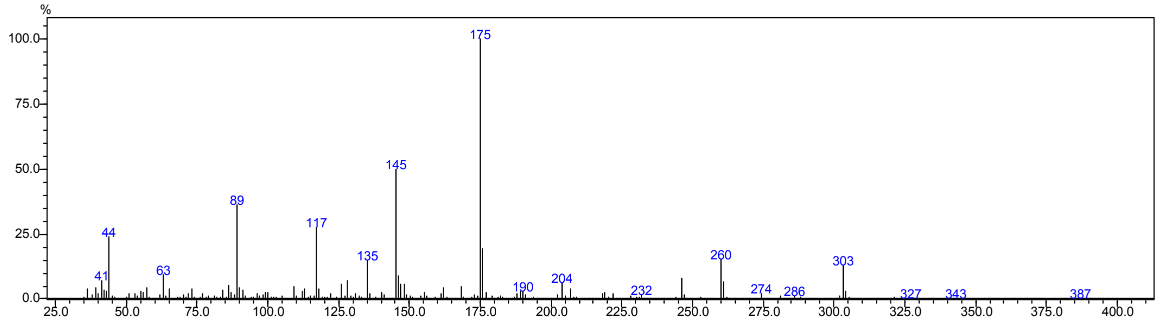

Fig S31 EI spectrum of **4b** (2*E*)-3-(2H-1,3-benzodioxol-5-yl)-*N,N*-dibutylprop-2-enamide

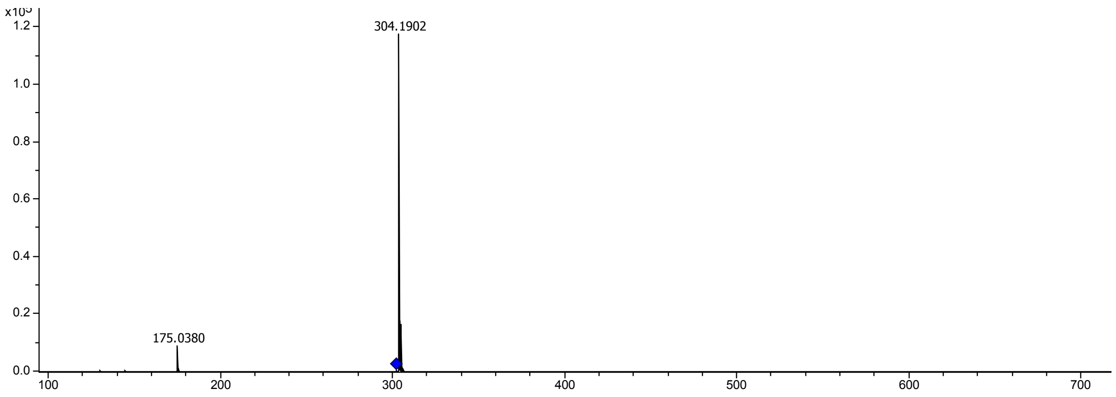

Fig S32 HRESI spectrum of **4b** (2*E*)-3-(2H-1,3-benzodioxol-5-yl)-*N,N*-dibutylprop-2-enamide

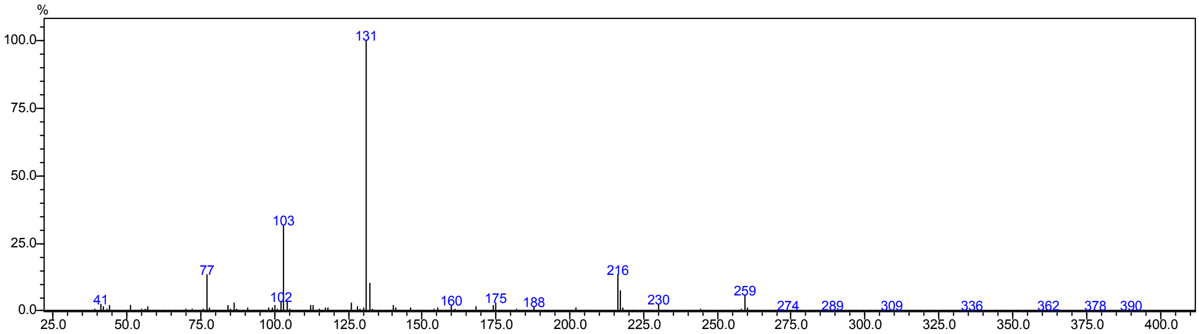

Fig S33 EI spectrum of **5a** (2*E*)-*N,N*-dibutyl-3-phenylprop-2-enamide

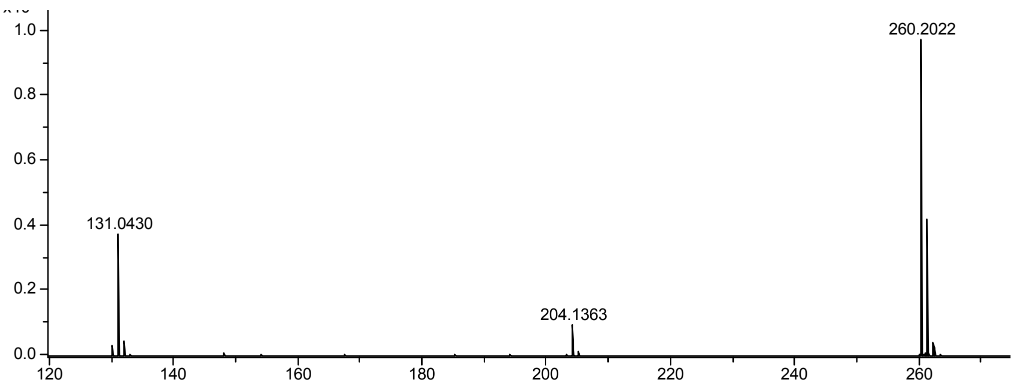

Fig S34 HRESI spectrum of **5a** (2*E*)-*N,N*-dibutyl-3-phenylprop-2-enamide

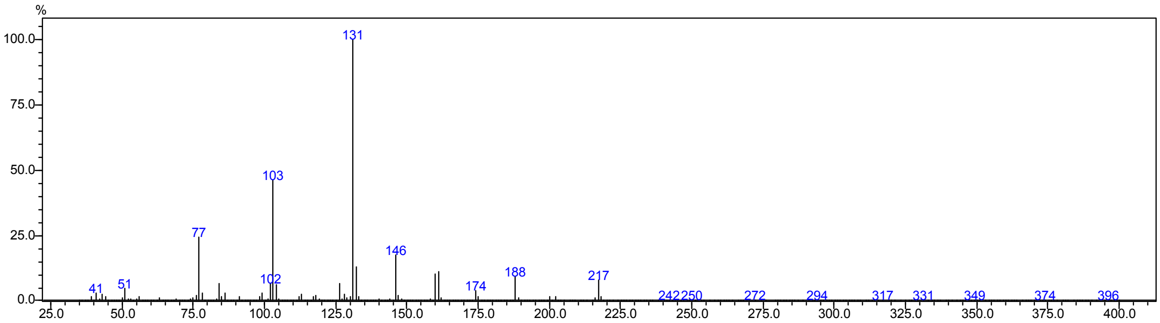

Fig S35 EI spectrum of **5b** (2*E*)-*N*-pentyl-3-phenylprop-2-enamide

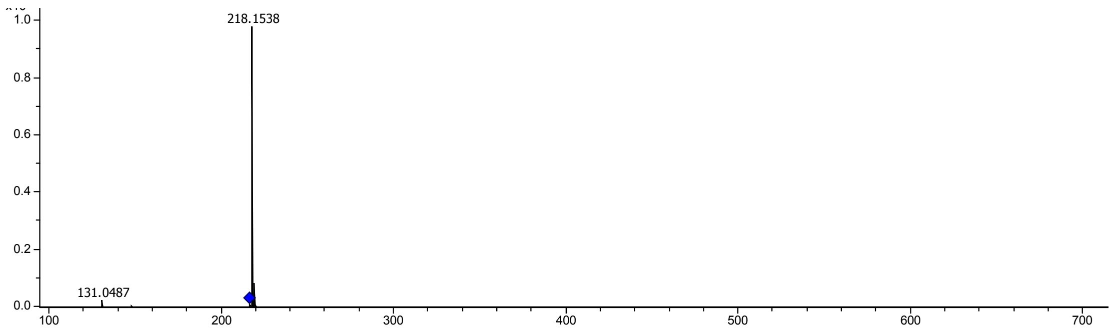

Fig S36 HRESI spectrum of **5b** (2*E*)-*N*-pentyl-3-phenylprop-2-enamide

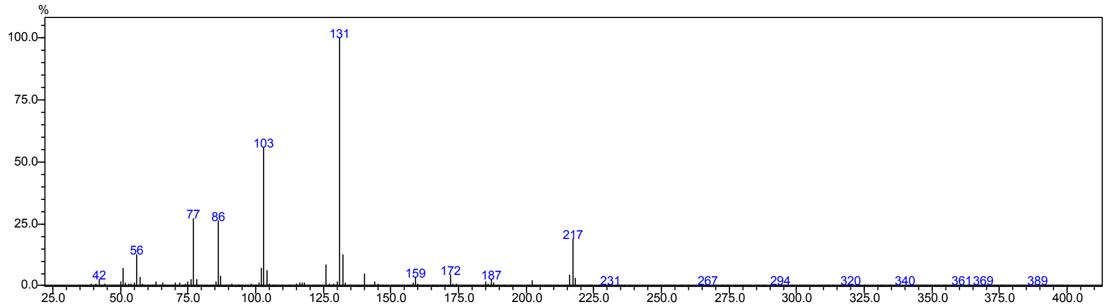

Fig S37 EI spectrum of **5c** (2*E*)-1-(morpholin-4-yl)-3-phenylprop-2-en-1-one

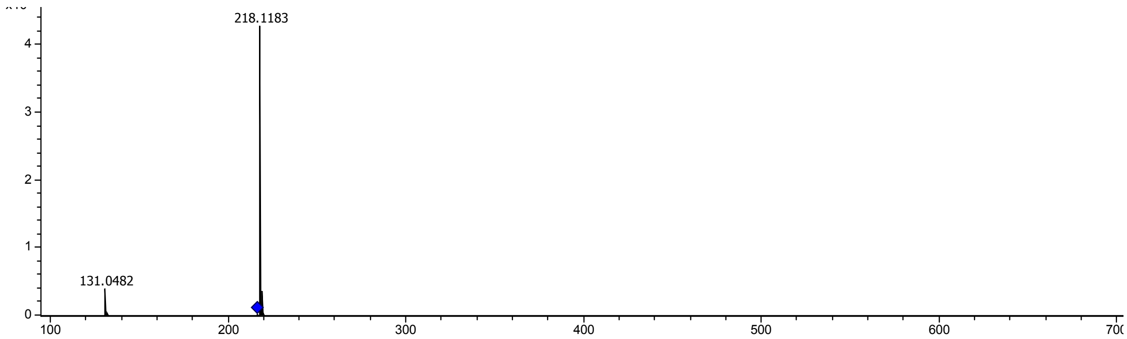

Fig S38 HRESI spectrum of **5c** (2*E*)-1-(morpholin-4-yl)-3-phenylprop-2-en-1-one

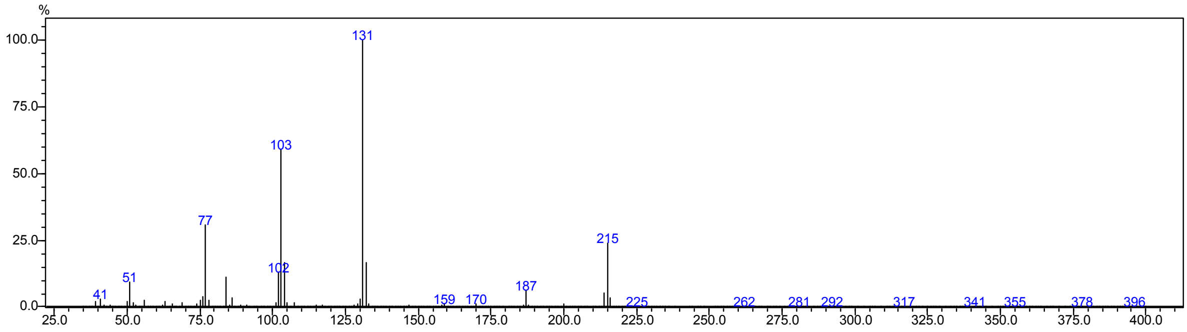

Fig S39 EI spectrum of **5d** 1-[(2*E*)-3-phenylprop-2-enoyl]pyrrolidin-2-one

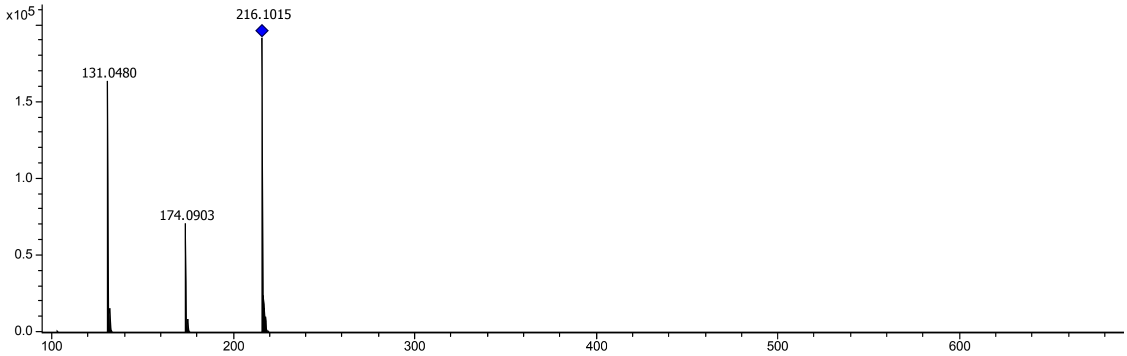

Fig S40 HRESI spectrum of **5d** 1-[(2*E*)-3-phenylprop-2-enoyl]pyrrolidin-2-one

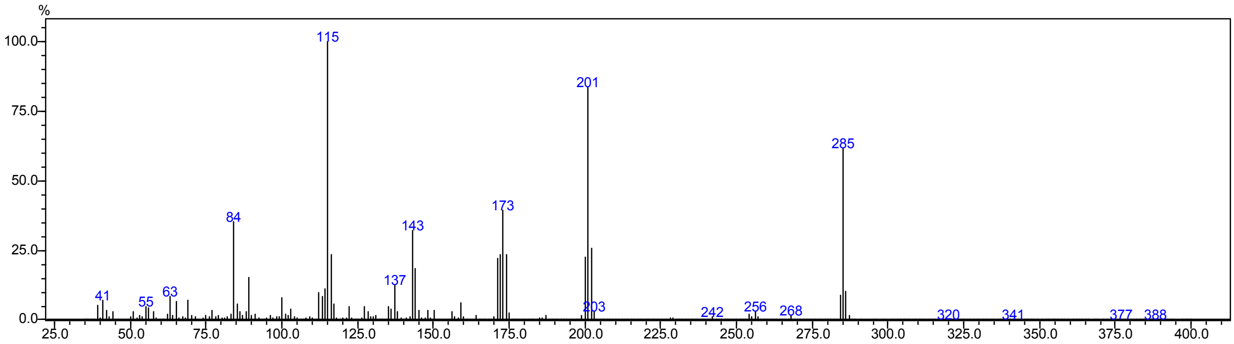

Fig S41 EI spectrum of **6a** (2*E*,4*E*)-5-(2*H*-1,3-benzodioxol-5-yl)-1-(piperidin-1-yl)penta-2,4-dien-1-one

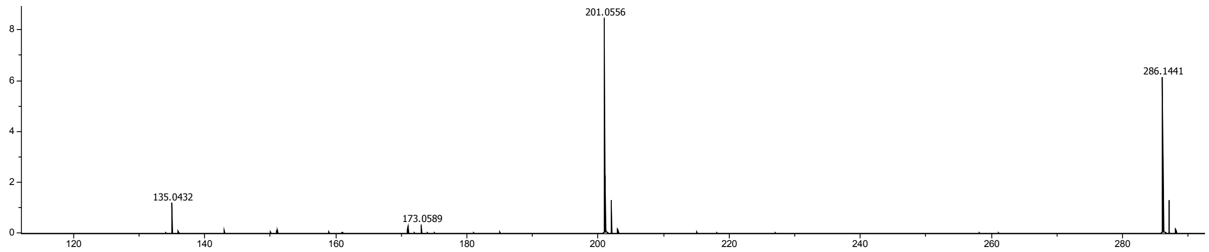

Fig S42 HRESI spectrum of **6a** (2*E*,4*E*)-5-(2*H*-1,3-benzodioxol-5-yl)-1-(piperidin-1-yl)penta-2,4-dien-1-one

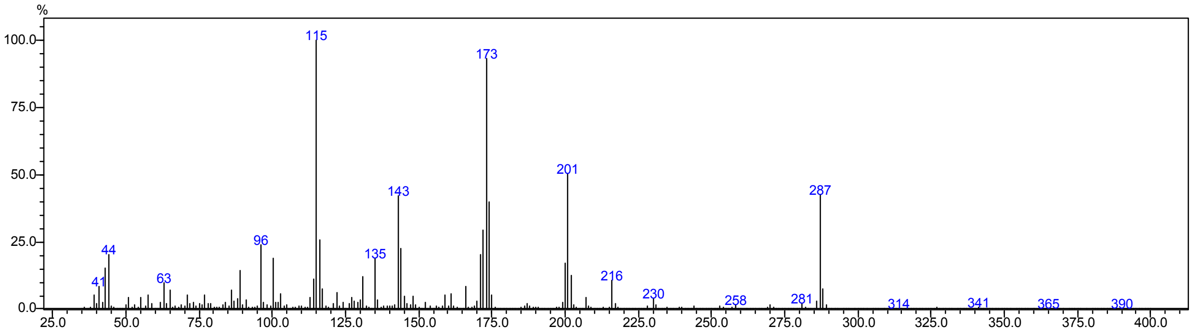

Fig S43 EI spectrum of **6b** (2*E*,4*E*)-5-(2*H*-1,3-benzodioxol-5-yl)-*N*-pentylpenta-2,4-dienamide

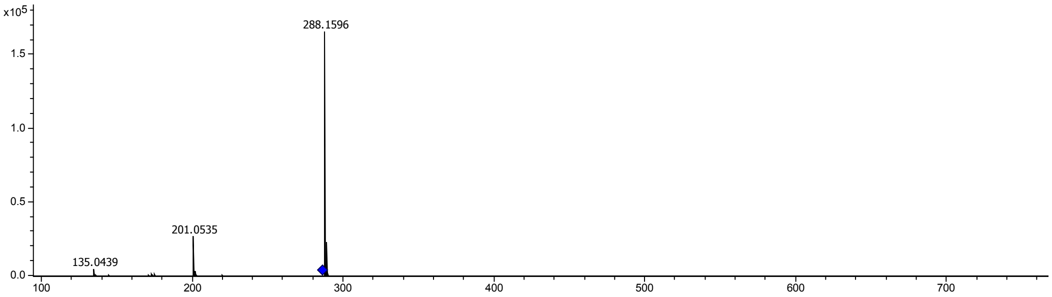

Fig S44 HRESI spectrum of **6b** (2*E*,4*E*)-5-(2*H*-1,3-benzodioxol-5-yl)-*N*-pentylpenta-2,4-dienamide

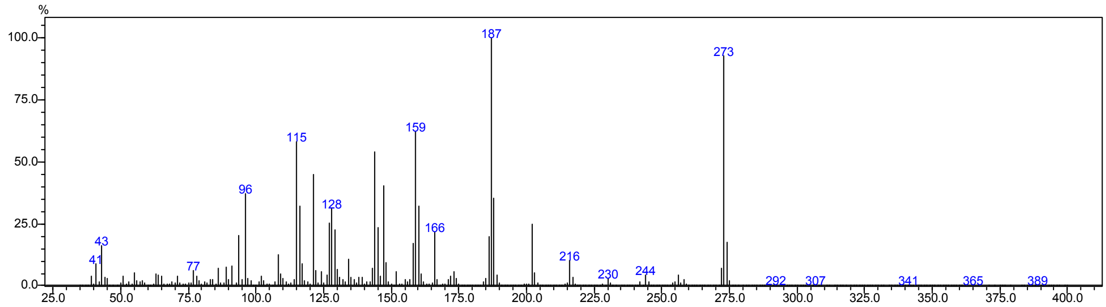

Fig S45 EI spectrum of **7a** (2*E*,4*E*)-5-(4-methoxyphenyl)-*N*-pentylpenta-2,4-dienamide

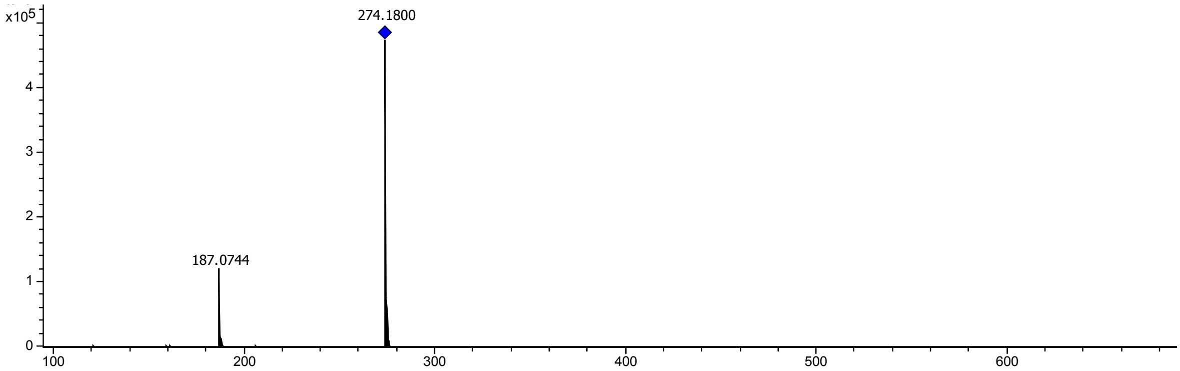

Fig S46 HRESI spectrum of **7a** (2*E*,4*E*)-5-(4-methoxyphenyl)-*N*-pentylpenta-2,4-dienamide

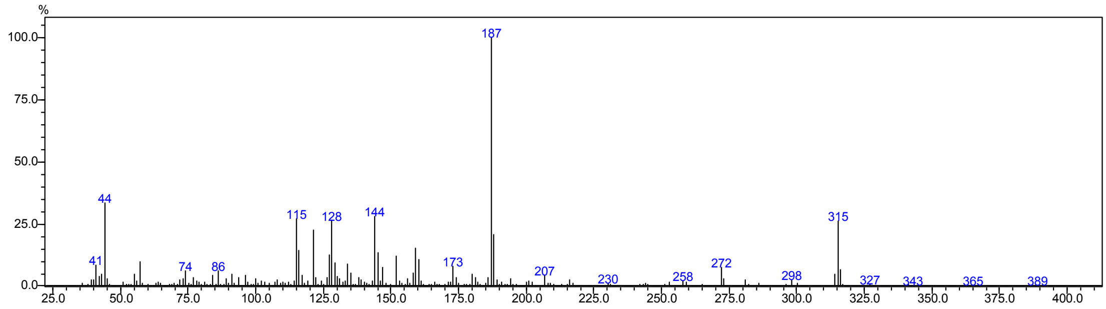

Fig S47 EI spectrum of **7b** (2*E*,4*E*)-*N,N*-dibutyl-5-(4-methoxyphenyl)penta-2,4-dienamide

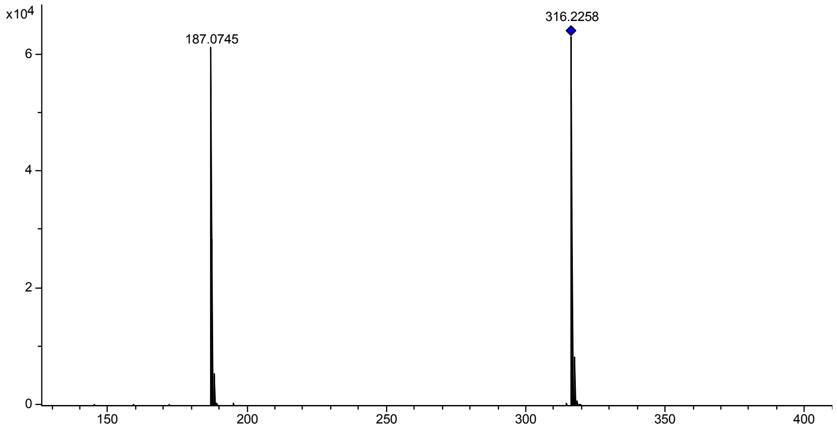

Fig S48 HRESI spectrum of **7b** (2*E*,4*E*)-*N,N*-dibutyl-5-(4-methoxyphenyl)penta-2,4-dienamide

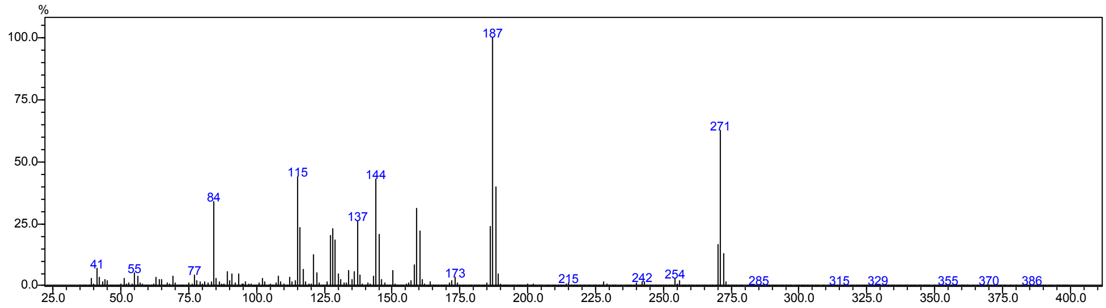

Fig S49 EI spectrum of **7c** (2*E*,4*E*)-5-(4-methoxyphenyl)-1-(piperidin-1-yl)penta-2,4-dien-1-one

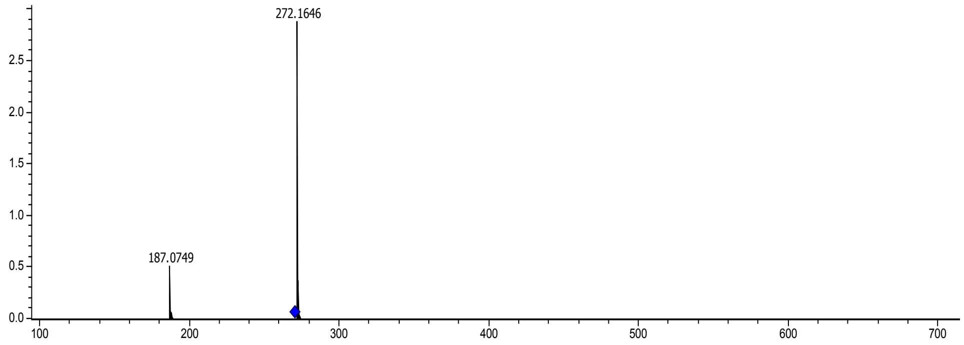

Fig S50 HRESI spectrum of **7c** (2*E*,4*E*)-5-(4-methoxyphenyl)-1-(piperidin-1-yl)penta-2,4-dien-1-one

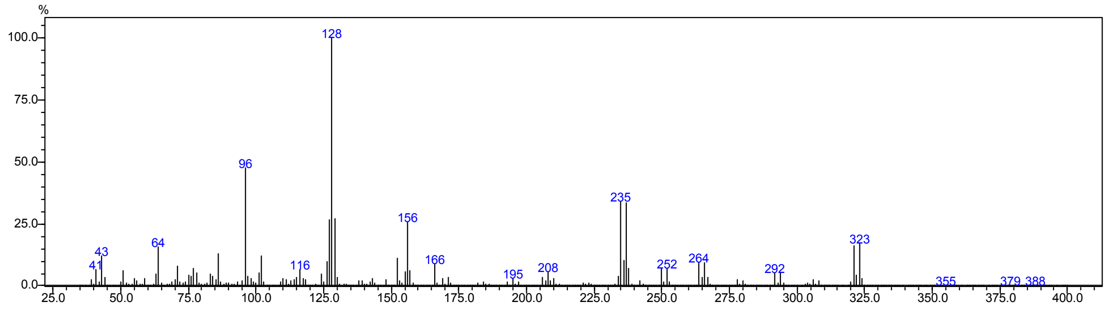

Fig S51 EI spectrum of **8a** (2*E*,4*E*)-5-(4-bromophenyl)-*N*-pentylpenta-2,4-dienamide

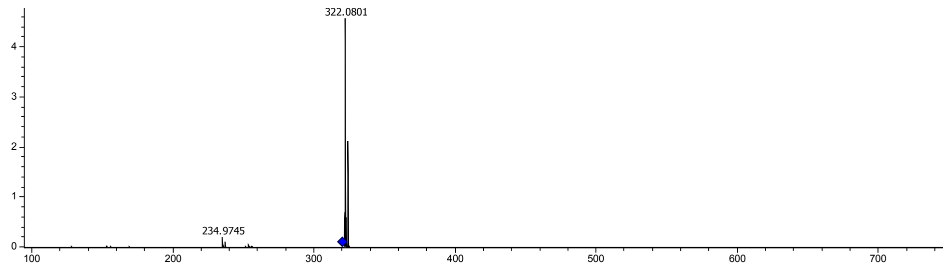

Fig S52 HRESI spectrum of **8a** (2*E*,4*E*)-5-(4-bromophenyl)-*N*-pentylpenta-2,4-dienamide

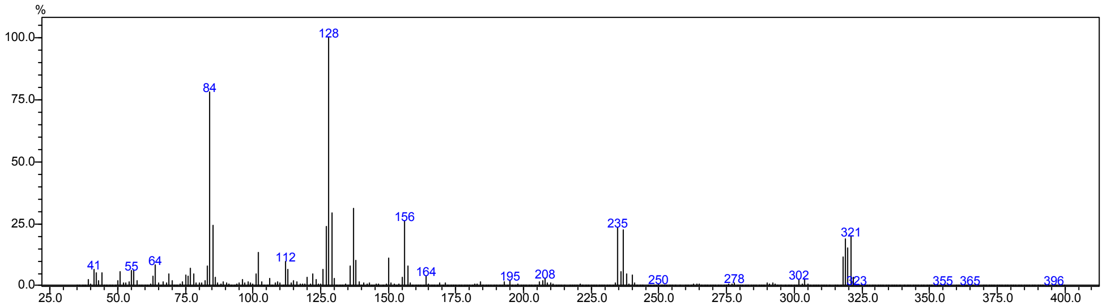

Fig S53 EI spectrum of **8b** (2*E*,4*E*)-5-(4-bromophenyl)-1-(piperidin-1-yl)penta-2,4-dien-1-one

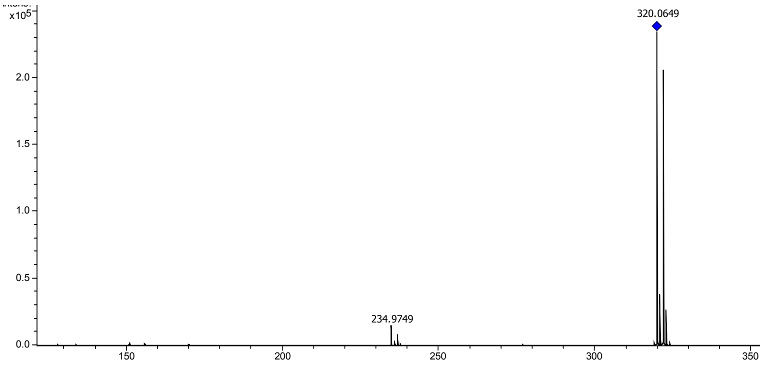

Fig S54 HRESI spectrum of **8b** (2*E*,4*E*)-5-(4-bromophenyl)-1-(piperidin-1-yl)penta-2,4-dien-1-one

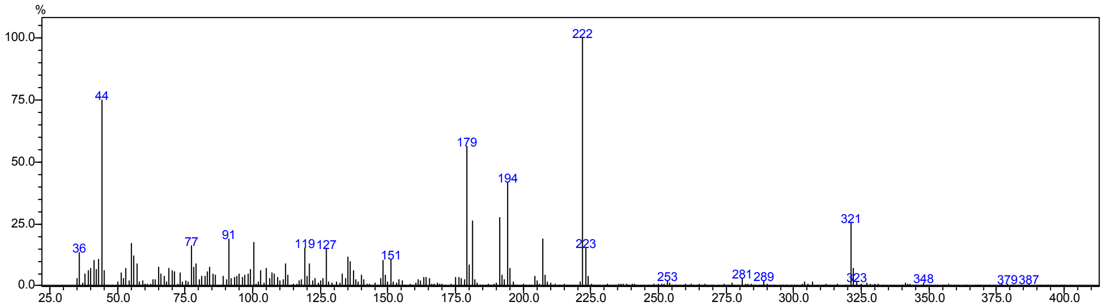

Fig S55 EI spectrum of **9** 1-[3-(3,4,5-trimethoxyphenyl)propanoyl]piperidin-2-one

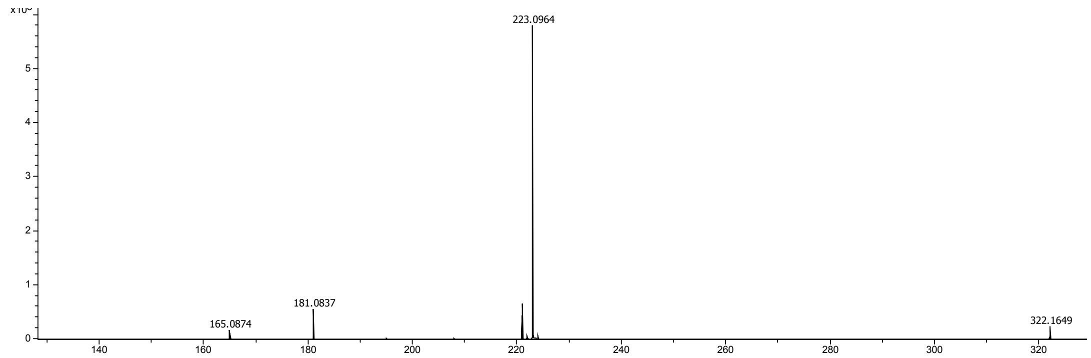

Fig S56 HRESI spectrum of **9** 1-[3-(3,4,5-trimethoxyphenyl)propanoyl]piperidin-2-one

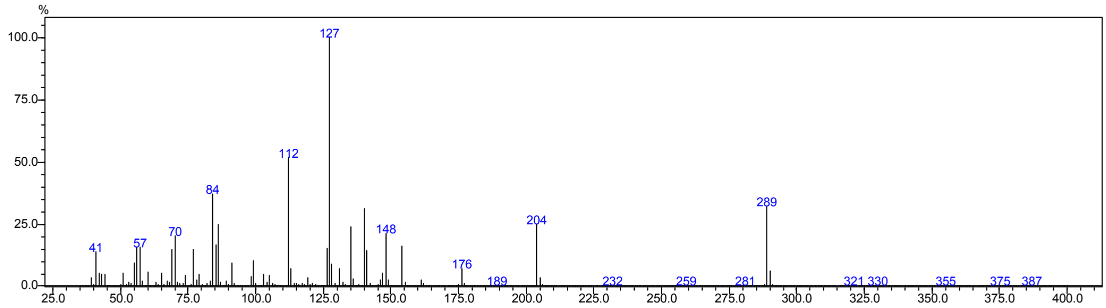

Fig S57 EI spectrum of **10** (2*E*,4*E*)-5-(2*H*-1,3-benzodioxol-5-yl)-1-(piperidin-1-yl)penta-2,4-dien-1-one

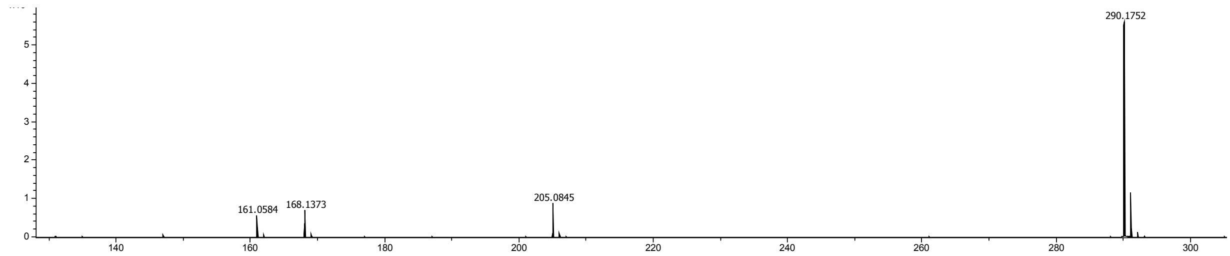

Fig S58 HRESI spectrum of **10** (2*E*,4*E*)-5-(2*H*-1,3-benzodioxol-5-yl)-1-(piperidin-1-yl)penta-2,4-dien-1-one
